# Network models of prostate cancer immune microenvironments identify ROMO1 as heterogeneity and prognostic marker

**DOI:** 10.1038/s41598-021-03946-w

**Published:** 2022-01-07

**Authors:** Lei Wang, Xudong Liu, Zhe Liu, Yafan Wang, Mengdi Fan, Jinyue Yin, Yu Zhang, Ying Ma, Jia Luo, Rui Li, Xue Zhao, Peiju Zhang, Lijun Zhao, Jinke Fan, Yuxuan Chen, Wei Lu, Xinqiang Song

**Affiliations:** 1 College of Life Sciences, Xinyang Normal University, Xinyang, 464000 China; 2 College of Medicine, Xinyang Normal University, Xinyang, 464000 China; 3 Department of Computer Science, City University of Hong Kong, Hong Kong, China; 4 Department of Recovery Medicine, People’s Liberation Army 990 Hospital, Xinyang, China; 5 Department of Urology, Central Hospital of Xinyang, Xinyang, China

**Keywords:** Cancer therapy, Tumour biomarkers, Tumour heterogeneity, Diagnostic markers, Genetics, Biomarkers, Diseases

## Abstract

Prostate cancer (PCa) is the fifth leading cause of death from cancer in men worldwide. Its treatment remains challenging due to the heterogeneity of the tumor, mainly because of the lack of effective and targeted prognostic markers at the system biology level. First, the data were retrieved from TCGA dataset, and valid samples were obtained by consistent clustering and principal component analysis; next, key genes were analyzed for prognosis of PCa using WGCNA, MEGENA, and LASSO Cox regression model analysis, while key genes were screened based on disease-free survival significance. Finally, TIMER data were selected to explore the relationship between genes and tumor immune infiltration, and GSCAlite was used to explore the small-molecule targeted drugs that act with them. Here, we used tumor subtype analysis and an energetic co-expression network algorithm of WGCNA and MEGENA to identify a signal dominated by the *ROMO1* to predict PCa prognosis. Cox regression analysis of *ROMO1* was an independent influence, and the prognostic value of this biomarker was validated in the training set, the validated data itself, and external data, respectively. This biomarker correlates with tumor immune infiltration and has a high degree of infiltration, poor prognosis, and strong correlation with CD8+T cells. Gene function annotation and other analyses also implied a potential molecular mechanism for *ROMO1*. In conclusion, we putative *ROMO1* as a portal key prognostic gene for the diagnosis and prognosis of PCa, which provides new insights into the diagnosis and treatment of PCa.

## Introduction

Prostate cancer (PCa), an epithelial malignancy of the prostate, is the second most common cancer in the world and one of the most common malignancies in men^1,2^. At present, PCa has become a global public problem threatening human health and life^3^, with more than 1,275,000 new diagnoses and 350,000 deaths each year. The factors associated with the development of PCa are mainly involved in age, race, family heritage, geography, and diet^4–6^. According to the Epidemiology and End Results report (https://seer.cancer.gov//), the five-year survival rate for PCa is 97.5%^7^, and it will account for 13.1% of all new cancers in 2021. Although there are many types of research on PCa, its etiology and pathogenesis remain not fully understood. The current treatments for the PCa include radical surgery^8^, external beam radiotherapy^7^, brachytherapy^9^, local treatment of experimental PCa^10^, endocrine therapy^11^, and chemotherapy^12^, etc. Serum prostate-specific antigen (PSA) is now commonly used as the preferred marker for screening PCa, with low specificity^13^. The immune system plays a huge role in preventing and treating of tumors^14^. Treatments developed to target the immune system, such as cancer vaccines, can effectively target specific antigens expressed on the surface of cancer cells, thereby protecting normal cells from drug damage^15^. However, due to the heterogeneity within tumors and because cancer vaccines target only a limited number of specific antigens, cancer cells that do not express these antigens or a group of cancer cells that have mutations that result in altered surface antigens can evade immune control and thus create new tumor populations that can resist treatment with vaccines encoding the same tumor-associated antigens^16,17^. Therefore, the development of new effective oncology treatments against heterogeneous populations of tumors has become an urgent task. However, immunotherapy for PCa has not been efficacious for patients in the past^18^. Therefore, comprehensive bioinformatics screening for new diagnostic and prognostic indicators and the proposal of new effective cancer drugs are of great importance for the treatment and diagnosis of PCa.

Intratumoral heterogeneity is a vital feature of tumorigenesis^19^. As cancer cells divide and proliferate, somatic mutations accumulate, some of which give cancer cells a more significant adaptive advantage^20–23^. Meanwhile, alterations in epigenetics with the tumor microenvironment may lead to different molecular subtypes that have varying tissue types, invasive capabilities, and degrees of differentiation^24–27^. The presence of heterogeneity makes tumor treatment very challenging, while the discovery of tumor driver genes and associated drugs targeting these genes offers the possibility of treating these tumors^28,29^. A comprehensive analysis of 333 cases of primary prostate cancer has identified three molecular subtypes^30^. Precision medicine for PCa needs to consider different molecular subtypes so that tumor heterogeneity can be achieved and patients can be offered personalized treatment options.

Reactive oxygen species (ROS) modulator 1 (ROMO1) is a membrane protein found in mitochondria that is important for regulating mitochondrial ROS production and redox sensing^31^. Romo1 is capable of triggering and exacerbating cancer through extracellular signal-regulated kinases (ERKs) and nuclear factor-kB (NF-kB)-induced reactive oxygen species (ROS)^32^. ROS can trigger and exacerbate cancer in a variety of malignancies^33,34^. It can also influence cancer cell invasion by affecting the epithelial-mesenchymal transition (EMT) pathway^35,36^. This protein affects signaling pathways and ROS homeostasis, affects the G2/M phase cell cycle, and leads to cell overgrowth through increased expression levels^37^. Nevertheless, ROMO1 has not been reported in the tumor microenvironment as well as in the development of prostate cancer.

With the rapid development of next-generation sequencing technologies, bioinformatics analysis allows us to understand tumor characterizes at a multi-omics level^38^. Research in RNA sequencing (RNA-seq), Co-expression network analysis, immunohistochemistry, immuno-infiltration, and targeted drug studies, etc., are now widely used. RNA sequencing can study the transcriptional profiles of cell populations or the average expression levels of tissues^39^. The Cancer Genome Atlas (TCGA)^40^, Gene Expression Profiling Interactive Analysis (GEPIA)^41^, The Human Protein Atlas (HPA)^42^, etc. are open databases for the collection and storage of patient genomic data. This study explores the PCa RNA-seq data to discover new biomarkers and provide new insights for the diagnosis and treatment of PCa.

## Materials and methods

### Data acquisition

Gene expression profiles and clinical data for PCa were downloaded using the UCSC Xena (http://xena.ucsc.edu/) database^43^, containing 436 PCa tissue and 114 normal tissues. Patients with unclear survival time and survival status characteristics were excluded from the cohort, and all data were collected on 10 April 2021.

### Determination of cluster count and membership

Based on the expression profile of PCa, ConensusClusterPlus (v. 1.52.0) R language software package has carried out unsupervised clustering classification for 550 samples^44,45^. The project selection criteria were selected 1000 iterations and 80% resampling rate Pearson correlation. Concurrently, principal component analysis for further filtering of the results of consensus clustering is employed. Reshape2 (v. 1.4.4) and factoextra (v. 1.0.7) two R language software packages were used to reduce the dimensions of clusters of different clusters to verify the results, and the first two principal components of the principal component analysis were selected for subsequent analysis. Also, all samples are within the cluster and within the cutoff height of (− 100 ~ 150) and cutoff width of (− 200 ~ 150). The ggplot2 (v. 3.3.3) R language software package was used to draw the graph to display the results.

### Identification of differentially expressed genes based on consensus clustering and PCA

The Limma (v. 3.46.0) R language package was used to screen differentially expressed genes (DEGs) among noncancerous samples with different prostate carcinoma subtypes^46^. The DEGs are identified by significant cutoffs. Genes with |log2FC| (fold change) ≥ 1 and *P-value* ≤ 0.01 is considered as DEGs. The R language software packages of ggplot2 (v. 3.3.3) and RColorBrewer (v. 1.1.2) were used to draw the graph to display the results. The Venn diagram of the overlapping DEGs was outputted by the Draw Venn diagram website (http://bioinformatics.psb.ugent.be/webtools/Venn/), and the Upset plot was drawn by the UpSetR R language package.

### Functional enrichment analysis of noncancerous samples and different tumor subtypes of DEGs

To elucidate the biological functions of the DEGs, we performed further functional annotation and pathway enrichment analysis using the Metascape (https://metascape.org) online tools^47^. *P-value* < 0.05 was considered as the cutoff value. An R package heatmap is used in heatmap figure. And two-way hierarchical clustering is used for clustering.

### Gene co-expression network analysis to obtain key gene modules associated with tumorigenesis and malignant progression

Gene co-expression network analysis is widely used in identifying functional gene modules associated with human disease and has proven to be highly effective^48,49^. The complexity of tumors is further expressed through intratumoral heterogeneity and the tumor microenvironment, which encompasses intratumoral cell differences and interactions and the expression of several biomarkers between normal and different tumor cells^50,51^. In this study, we used Weighted Gene Co-expression Network Analysis (WGCNA) and Multiscale Embedded Gene Co-expression Network Analysis (MEGENA) to identify key genes associated with prostate carcinogenesis as well as tumor malignant progression WGCNA (v. 1.70-3) R language package was used to perform a WGCNA of all DEGs with clinical traits, mining modules, and key genes associated with clinical characteristics^52^:

1. The gene correlation matrix was transformed into a scale-free network.
2. Unsupervised hierarchical clustering was performed using a dynamic tree chopping algorithm, and the clustered tree branches were defined as modules.
3. The correlation between each module and clinical traits was calculated, and the modules with higher correlations were selected.

Subsequently, the hub genes in the selected modules were ranked by intra-module connectivity and correlation with module trait genes, and finally, candidate genes were identified.

MEGENA is an innovative co-expression network analysis method that offers unique advantages over WGCNA for efficiently constructing large-scale co-expression planar filter networks and preserving gene interactions^53^. The R software package MEGENA (v. 1.3.7) is used to perform MEGENA, which consists of the following steps: (1) constructing a planar filtered network (PFN); firstly, calculating correlation coefficients based on gene expression profiles, and then filtering and clustering gene pairs using a parallel filtering method to obtain a fast planar filter network; (2) multi-scale clustering analysis; from the initial PFN of the connected components, multi-scale clustering of each parent cluster can obtain more sub-modules, followed by hierarchical clustering results; (3) downstream analysis, using multiscale hub analysis (MHA) to identify important hubs based on the network topology; (4) Finally, the correlation between clustering results and clinical information was analyzed by cluster-trait association analysis (CTA). To test whether selected modules in WGCN and multiscale hub genes in MEGCN were highly associated with tumorigenesis and malignant progression, we performed GO and KEGG pathway^54^ analysis on all genes in the selected DAVID (https://david.ncifcrf.gov/) database^55^ for hub modules. *P* ≤ *0.05* was considered as the cut-off criterion for identifying enrichment. Similarly, an R package heatmap is used in heatmap figure. And two-way hierarchical clustering is used for clustering.

### Validation and survival analysis of pivotal genes

In WGCNA, when hub modules were identified, we selected candidate hub genes by module connectivity (cor. gene module-Membership (MM) > 0.8) and clinical feature correlation (cor.geneTraitSignificance (GS) > 0.2), both by Pearson correlation absolute values were used to determine. At the same time, we chose the STRING database (https://www.string-db.org/)^56^ to identify the connectivity of candidate genes. Next, Cytoscape's plug-in CytoHubba was used to identify top50 genes as candidate genes^57^. The gene modules from the WGCNA and MEGENA analyses are saved separately. The userListEnrichment R function was used to calculate the overlap of genes between the WGCNA and MEGENA modules. The WGCNA and MEGENA modules that overlapped significantly and were significantly associated with PCa were retained. In this study, we used the Gene Expression Profiling Interactive Analysis (GEPIA) dataset (http://gepia.cancer-pku.cn/) for prognostic analysis of PCa^58^. We analyzed the differential expression of key hub genes in PCa and their association with RFS using GEPIA. P-value and fold change were defined as 0.05 and group limit of 50% for two survival analyses. Also, normal distribution tests and differential analysis of hub genes in normal versus tumor tissue and normal versus different tumor subtypes were performed based on the Wilcoxon test. Finally, Human Protein Atlas Dataset (https://www.proteinatlas.org/)^59^ was used to validate the immunohistochemistry (IHC) of the hub gene.

### Development and validation of the hub gene prognostic model

To assess the prognostic value of hub genes, Cox regression analysis was used to evaluate the correlation between genes and survival status in a cohort of 494 PCas. Next, we chose the glmnet R language package for LASSO Cox regression modeling to narrow down the candidate genes and build prognostic models^60^. Ultimately, the screening retained three genes and their coefficients, and the penalty parameter λ was determined based on the minimum criterion. The algorithm obtained a more accurate model by constructing a penalty function. As a method for complex covariance data, variable selection can be achieved and parameter estimation to better address multiple covariances in regression analysis. The PCa expression data were centrally normalized using a scale function, and a risk score was calculated.

Patients with PCa OS were divided into low and high-risk subgroups based on median risk scores. Kaplan Meier analysis was performed to compare OS time assessment risk scores and overall survival between the two subgroups. To validate the accuracy and predictive power of the model, ROC curve analyses were performed using the survivor, survminer, and timeROC R packages for one year, three years, and five years, respectively, to calculate the area under The curve (AUC) and to compare the effect of the timeROC R package on classifier performance.

### Relationship between key genes and tumor-infiltrating immune cells

The Tumor Immunity Evaluation Resource (TIMER) (https://cistrome.shinyapps.io/timer/) is a comprehensive resource for detecting immune cell infiltration in tumor tissue using RNA-Seq expression profiling data and contains immune infiltration in different cancer types^61^. Hub gene expression was correlated with six types of immune infiltration (B cells, CD4 + T cells, CD8 + T cells, neutrophils, macrophages, and dendritic cells) were correlated and assessed using the Gene module. The SCNA module was also used to explore the correlation between somatic cell copy number changes and the abundance of immune infiltrates.

### Exploring the drug sensitivity of the hub gene

The GSCAlite (http://bioinfo.life.hust.edu.cn/web/GSCALite/) database is a genomic cancer analysis platform^62^. The database can be used for genomic and immunogenomic analyses. It also enables researchers to combine clinical information and small molecule drugs to mine candidate biomarkers and valuable small molecule drugs for better experimental design and further clinical trials. The GSCA data contains 33 cancer types from TCGA and normal tissue data from GTEx, and over 750 small-molecule drugs from GDSC and CTRP for 10,000 genomic data. Spearman correlations represent gene expression associated with drugs. A positive correlation means that a gene with high expression is resistant to the drug and vice versa.

### Statistical analysis

R^63^ (version 3.6.2) and related software packages were an application for all the statistical analyses. *P* < 0.05 is a statistically significant difference.

## Result

### Combined consistency clustering and principal component analysis to obtain sample cohorts

**Figure 1:**
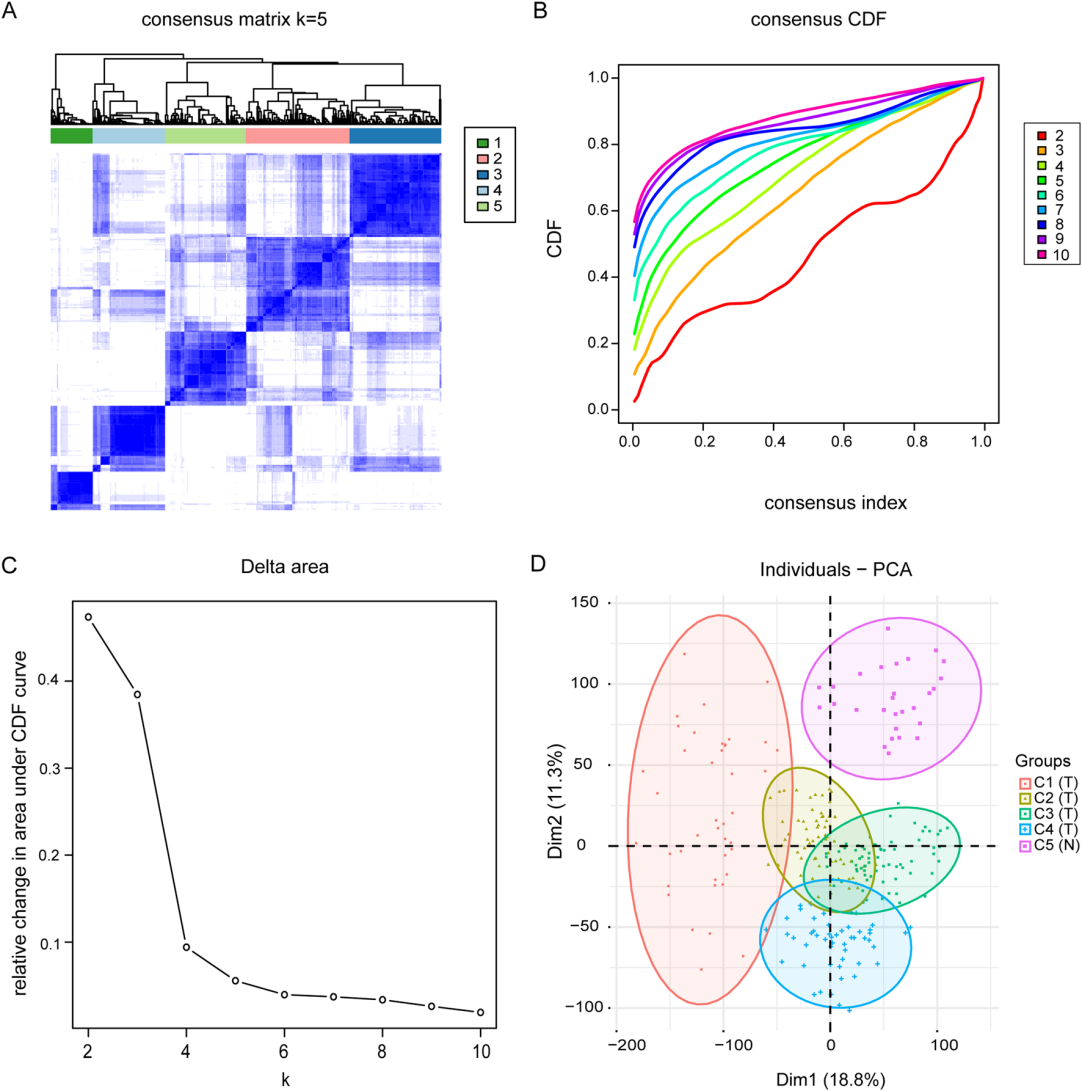
PCa tumor subtypes classification and identification in TCGA cohort. (**A**). The 550 samples were split into five clusters by the consensus clustering matrix (k = 5). (**B**).The consensus clustering approach established the cumulative distribution function (CDF) curve of Top 10. (**C**). The CDF Delta area curve of all samples with different k values. (**D**). Principal component analysis was carried out based on the results of consensus clustering.

The analytical process used in this study is shown in Fig. S1. To follow up on the molecular heterogeneity of PCa, we performed an unsupervised consensus analysis on all samples. In this study, we chose k = 5, which allowed us to divide all samples into five groups (Fig. S2). Among them, C1:59, C2:146, C3:129, C4:102, and C5:114. Moreover, we found that C1-C4 were tumor subgroups, and C5 were normal samples (Fig. 1A–C). Next, to verify the robustness of this classification, we performed another principal component analysis based on the results of consistent clustering and observed the subgroup differentiation. PC1 and PC2 were selected as the main components for the analysis, and 260 samples with significant differences were obtained after excluding outlier samples as well as those with insignificant differences (Table 1). As shown in Fig. 1D, there was an individual crossover between tumor subgroups, indicating good differentiation between subgroups. The clustering results were as follows: C1:41, C2:68, C3:72, C4:51, and C5:28, reflecting the impact of differences between tumor subgroups and normal samples on transcriptional profiles.

**Table 1:** Information on the clinical features of patients with prostate cancer.

Variable	Type	%
**PCA (260)**		
Tumor	232	89
Normol	28	11
**CCF**		
Cluster1	41	16
Cluster2	68	26
Cluster3	72	28
Cluster4	51	20
Cluster5	28	10
**Relaplse**		
NA	13	5
NO	206	79
YES	41	16
**Age**		
> = 60	163	63
< 60	97	37
**Gleason_score**		
6	17	6
7	144	55
8	28	11
9	69	27
10	2	1
**OS**		
0 < = OS < = 1000	142	55
1000 < OS < = 2000	84	32
2000 < OS < = 3000	24	9
3000 < OS < = 4000	5	2
4000 < OS < = 5000	3	1
OS > = 5000	2	1

### Identification of differentially expressed genes and GO functional annotation in PCa

**Figure 2:**
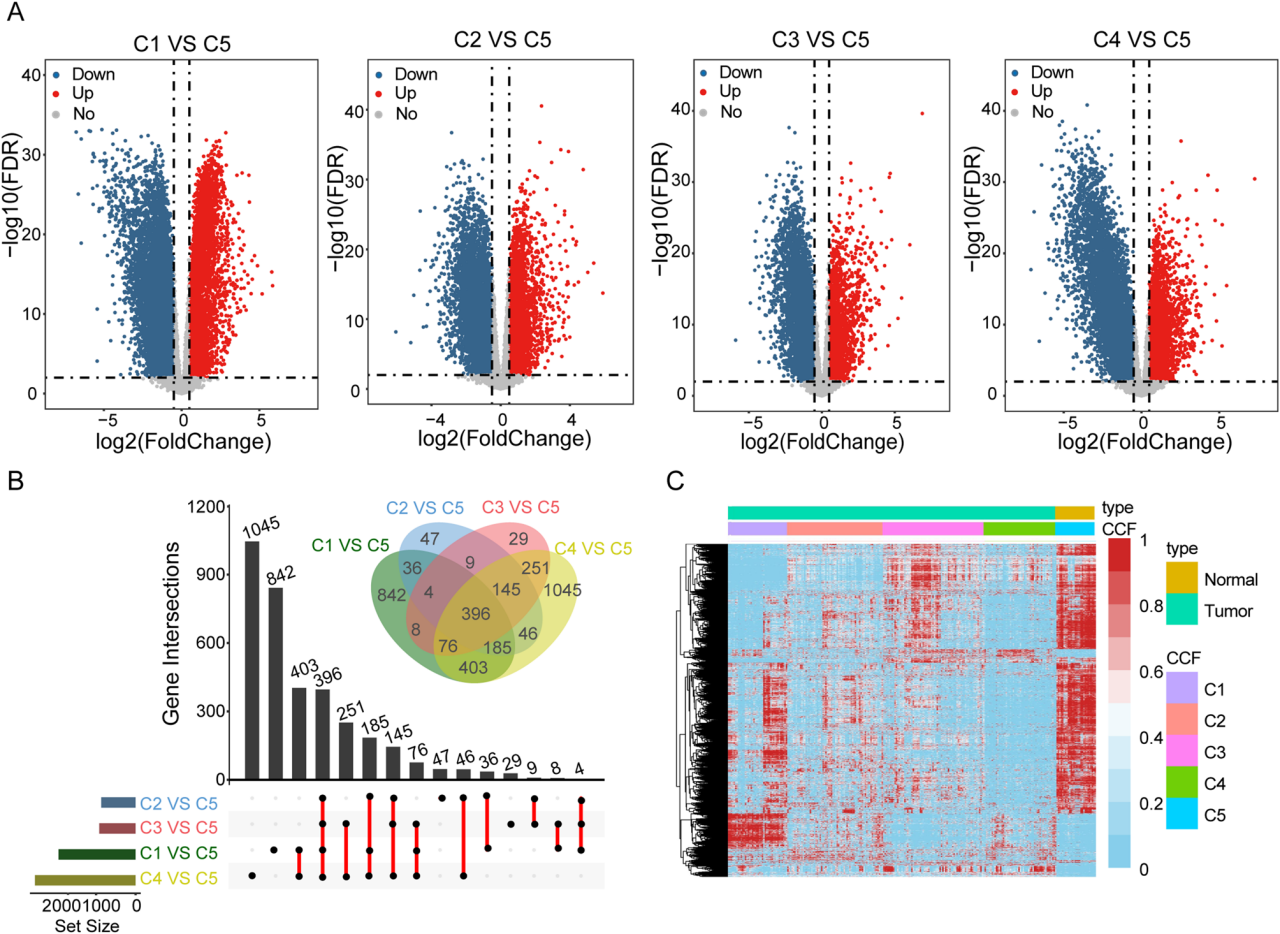
Analysis of differences between four tumor subtypes and normal samples. (**A**). Volcano plot of four groups, with |log2(FC)| > 2 and FDR < 0.01. (**B**) Venn diagram and Upset plot were used to visualize common genes between 4 clusters datasets. The number of genes annotated is presented on the y-axis. (**C**) The heat map shows the expression profiles of four groups of differentially expressed genes.

We performed differential gene expression analysis of the four tumor subgroups separately from normal samples. A total of 3521 differentially expressed genes were obtained. Of these, 581 up-regulated and 1432 down-regulated genes were obtained for C1 vs. C5, 231 up-regulated and 637 down-regulated genes were obtained for C2 vs. C5, 234 up-regulated and 684 down-regulated genes were obtained for C3 vs. C5, and 282 up-regulated and 2265 down-regulated genes were obtained for C4 vs. C5 (Fig. 2A). Comparisons between tumor subgroups revealed 842 DEGs specific to C1, 47 DEGs specific to C2, 29 DEGs specific to C3, and 1045 DEGs specific to C4 (Fig. 2B). Three hundred ninety-six overlap genes were also identified, suggesting a degree of similarity in expression profiles between tumor subgroups but also a high degree of heterogeneity between tumor subgroups (Fig. 2C).

### Functional enrichment analysis of four groups of differentially expressed genes

**Figure 3:**
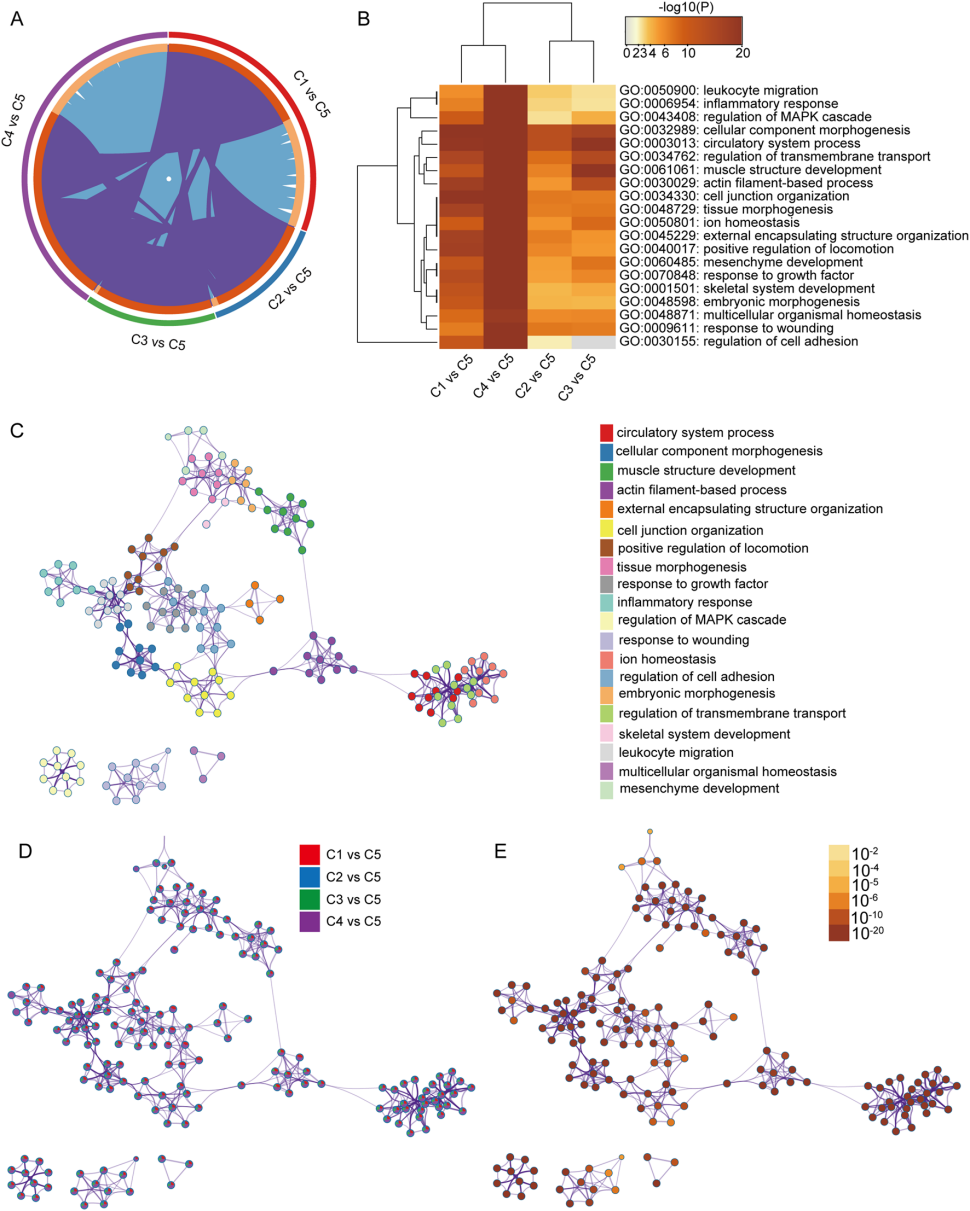
Meta-enrichment analysis summary for PFNs lists in 4 comparison cohorts. (**A**). The overlap of differentially expressed genes in the four selected groups is shown in the circus plot explored using Metascape. (**B**). Each box indicates whether the gene in each gene list (column, C1 vs C5, C4 vs C5, C2 vs C5, and C3 vs C5) enriched in each selected top 20 GO term. The darker the color, the more significance the *P*-value. R package heatmap is used in this process, and two-way hierarchical clustering is used for clustering. (**C**). The network of enriched terms is colored by cluster-ID, and nodes that share the same cluster are typically close to each other. (**D**). Network enrichment terms and genes are colored by the database, where the terms containing more genes tends to have more significates. (**E**). Coloring by the degree of enrichment, with darker colors indicating a greater number of genes enriched to that pathway or biological process class.

The researchers used the Metascape website to carry out a biological process analysis of the GO function of 3521 DEGs (Fig. S3). As shown in Fig. 3A, there was a strong interaction between the four groups of differentially expressed genes. Also, DEGs were found to be mainly enriched in functions such as leukocyte migration, inflammatory response, regulation of MAPK cascade, circulatory system processes cell component morphogenesis, and regulation of cell adhesion (Table 2). Moreover, Metascape enrichment analysis also showed that DEGs differed significantly between the four groups in the pathways regulating cell adhesion and leukocyte migration, demonstrating genetic and functional differences in tumor subtypes (Fig. 3B–E). Naturally, to ensure that the differential analysis did not filter out key genes, we also performed GSEA analysis on all genes and found that they were mainly enriched in P53, DNA repair, Notch signaling pathway, etc. (Fig. S4).

**Table 2:** Functional annotation of GO_BP for top20 of four sets of DEGs.

No	Category	Term ID	Description	LogP	Ratio
1	GO_BP	GO:0003013	Circulatory system process	− 71.68	207/611
2	GO_BP	GO:0032989	Cellular component morphogenesis	− 65.57	226/766
3	GO_BP	GO:0061061	Muscle structure development	− 56.30	186/609
4	GO_BP	GO:0030029	Actin filament-based process	− 55.21	215/794
5	GO_BP	GO:0045229	External encapsulating structure organization	− 52.90	143/398
6	GO_BP	GO:0034330	Cell junction organization	− 51.96	195/701
7	GO_BP	GO:0040017	Positive regulation of locomotion	− 48.52	171/587
8	GO_BP	GO:0048729	Tissue morphogenesis	− 46.92	177/638
9	GO_BP	GO:0070848	Response to growth factor	− 46.55	192/736
10	GO_BP	GO:0006954	Inflammatory response	− 45.06	196/778
11	GO_BP	GO:0043408	Regulation of MAPK cascade	− 44.56	185/712
12	GO_BP	GO:0009611	Response to wounding	− 41.77	172/658
13	GO_BP	GO:0050801	Ion homeostasis	− 40.81	190/787
14	GO_BP	GO:0030155	Regulation of cell adhesion	− 40.19	181/734
15	GO_BP	GO:0048598	Embryonic morphogenesis	− 39.35	154/569
16	GO_BP	GO:0034762	Regulation of transmembrane transport	− 38.50	152/565
17	GO_BP	GO:0001501	Skeletal system development	− 37.11	137/486
18	GO_BP	GO:0050900	Leukocyte migration	− 36.41	140/511
19	GO_BP	GO:0048871	Multicellular organismal homeostasi	− 35.28	139/516
20	GO_BP	GO:0060485	Mesenchyme development	− 34.80	99/288

### Co-expression network analysis reveals key modules of PCa network

**Figure 4:**
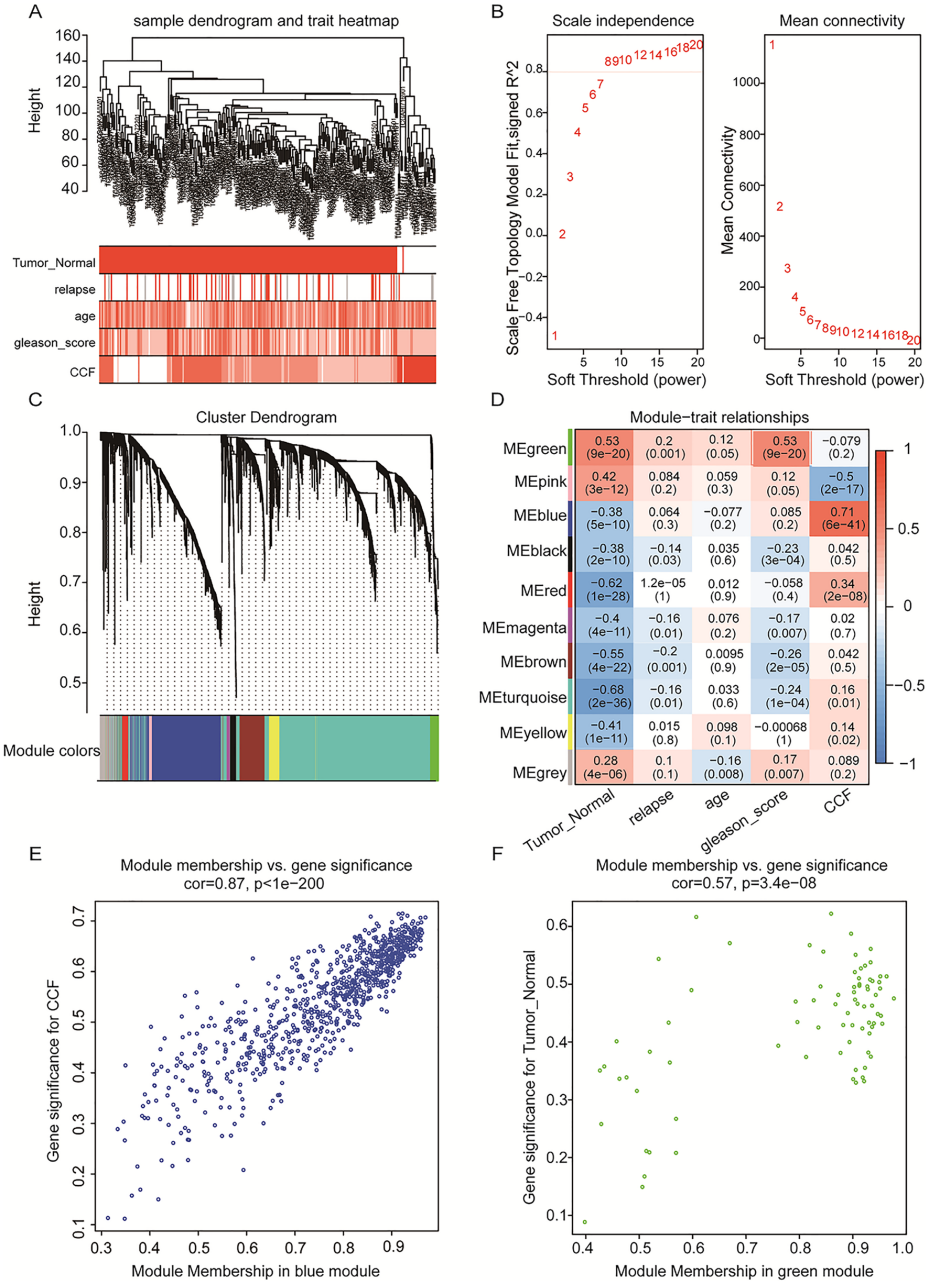
Analysis of weighted gene co-expression networks for the construction of PCa. (**A**). Dendrogram combining clinical data and transcriptional expression profiles for sample Euclidean distance clustering. (**B**). Analysis of soft threshold power for WGCNA: (left) Analysis of the scale-free matching index (β) for various soft threshold powers. (Right) Analysis of the average connectivity of various soft threshold powers. (**C**). Dendrogram of all differentially expressed genes clustered based on the measure of variability (1-TOM). The colors bands show the results obtained from the automated individual cluster analysis. (**D**). The heat map shows the relationship between MEs and clinical characteristics. Each cell contains the corresponding correlation coefficient and p-value. (**E**, **F**). Scatterplot of gene significance versus module affiliation in the three selected key modules blue, green modules.

**Figure 5:**
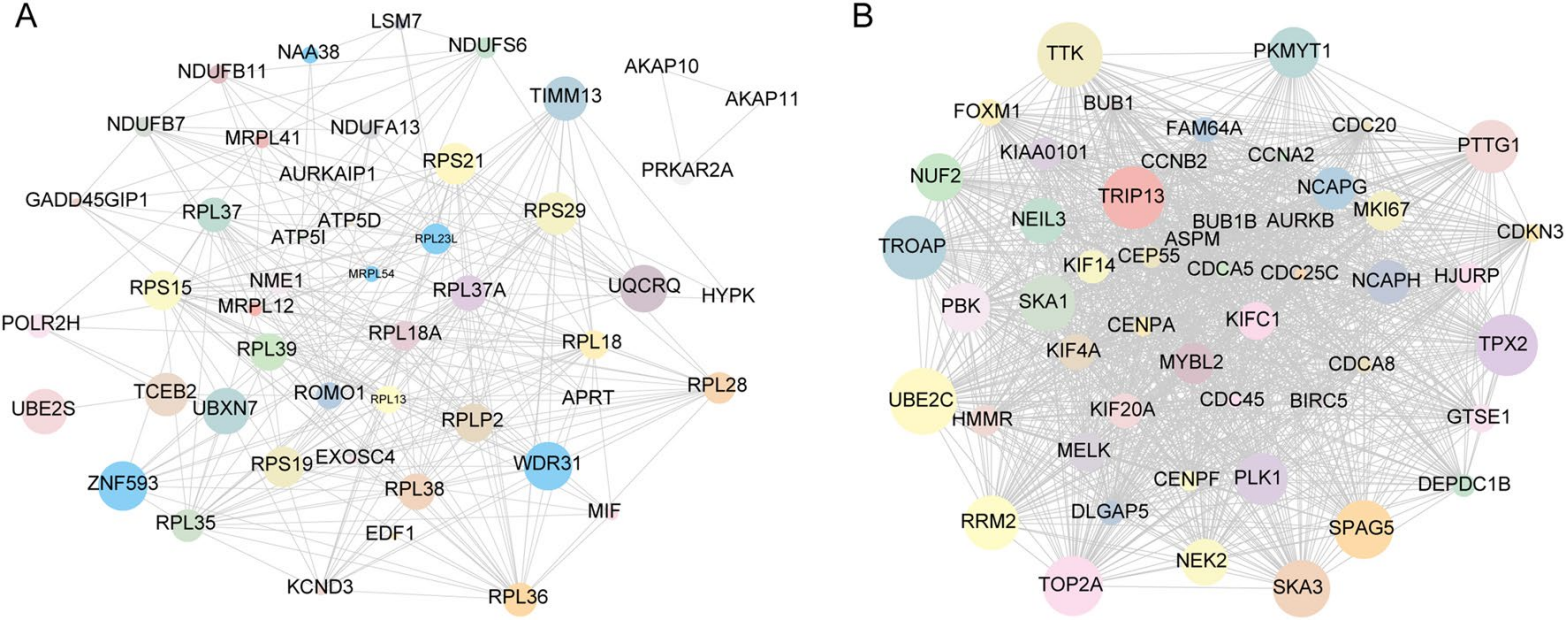
Weighted gene co-expression network analysis of gene interaction networks in key gene modules of PCa (**A**). The top 50 genes with the highest levels of intramodular connectivity in the blue module. (**B**). The top 50 genes with the highest levels of intramodular connectivity in the green module. (The size of the circle represents combined scores).

We used the expression profiles and clinical information of 260 samples from PCa as input files for WGCNA by integrating them. We eliminated two outlier samples based on the clustering tree and then plotted the sample dendrograms and expression heat maps of the traits (Fig. 4A). After excluding the two outlier samples, the remaining 258 samples and 3521 differentially expressed genes were subjected to WGCNA analysis (Fig. S5). We chose a soft threshold power of 8 (scale-free R^2^ = 0.85) by calculation, ensuring that the scale-free network was reasonable (Fig. 4B). We built the network by choosing the soft threshold. And the minimum number of genes in the modules was required to be 50. Subsequently, the initial modules were divided using a dynamic tree, and the genes were divided into modules based on the similarity of the characteristic genes in the gene clustering map, and ten gene modules were finally identified (Fig. 4C). One color indicates a gene module, while grey gene modules are considered invalid genes that cannot be assigned to a module. Next, we created a heat map of the correlation between gene modules and clinical information (Fig. 4D). We found that the blue module was found to have the highest correlation coefficient with consensus clustering (Pearson cor = 0.87, *p* = 1e−200) (Fig. 4E). This suggests that the genes in this module correlate with tumor typing as well as malignancy; meanwhile, we found a large correlation coefficient between the green module and tumorigenesis (Pearson cor = 0.57, *p=* 3e−08) (Fig. 4F). In contrast, the absolute values of the correlation coefficients between genes and age, recurrence status, and gleason_score were smaller in the other modules, suggesting that the genes are less relevant to other clinical information. Finally, we calculated the connectivity of the genes within the blue (Fig. 5A) and green modules (Fig. 5B) and selected the top50 most closely connected genes for subsequent analysis.

Meanwhile, the expression profiles of 3521 differentially expressed genes (DEGs) were applied to the multiscale embedded gene co-expression network (MEGENA) analysis. After parallelization, early termination, and pre-quality control steps, 10,333 gene pairs of PFNs were used to enter 1755 MCA for multiscale cluster identification. A total of 5 different scales were identified by multi-scale cluster analysis, and 226 significant modular clusters were constructed (Fig. S6), where the modular p-values were all less than 0.05.

**Figure 6:**
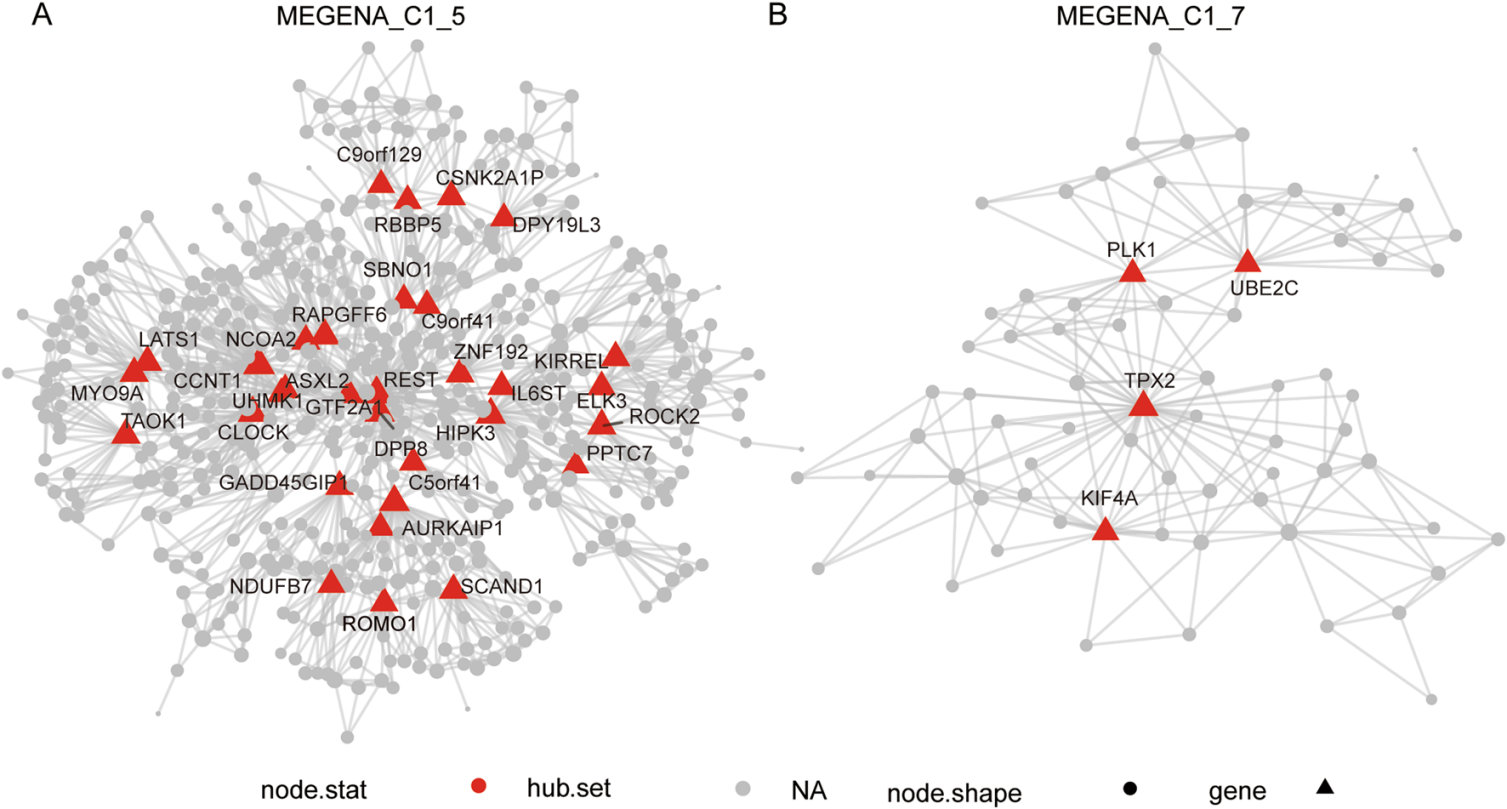
Visualization of interest modules and hub genes multiscale embedded gene co-expression network analysis (MEGENA). (**A**). Intergenic connectivity of genes in MEGENA_C1_5 and identification of key genes. (**B**). Intergenic connectivity of genes in MEGENA_C1_7 and identification of key genes. The red triangle represents the HUB gene and the gray circle represents the non-core gene.

**Figure 7:**
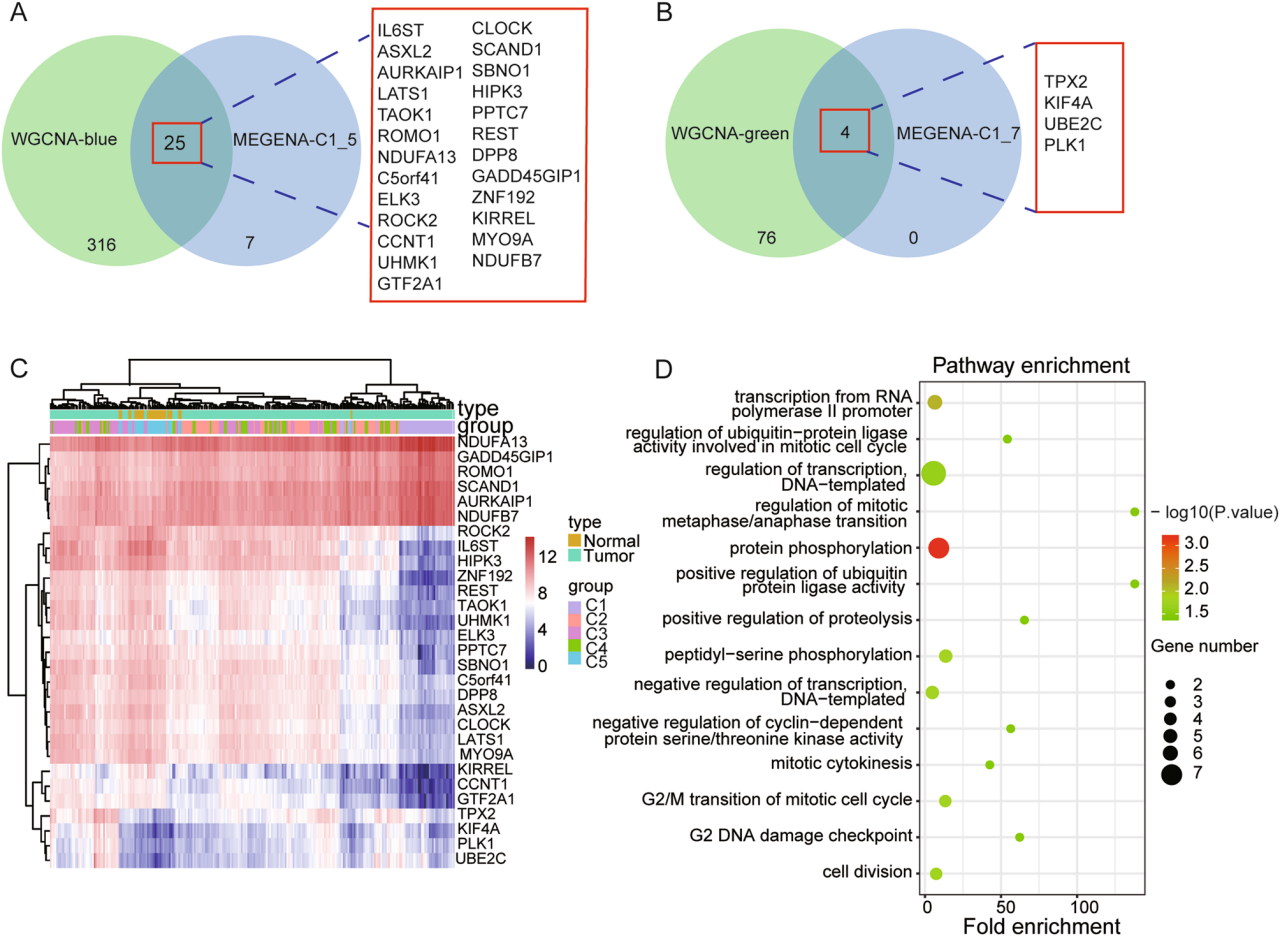
Visualization and functional annotation of candidate genes. Venn diagram showing all identified hub genes and their overlap in WGCNA-blue and MEGENA-C1_5 (**A**), and WGCNA-green and MEGENA-C1_7 (**B**). (**C**). Each box indicates the expression level of each candidate gene (row) in each sample (column). The red indicates the high expression, while the blue indicates the low expression. Similarly, an R package heatmap is used in this figure. And two-way hierarchical clustering is used for clustering. (**D**). GO enrichment analysis was performed on the candidate genes.

Comparing the significant modules of WGCNA with the MEGENA modules, we found a degree of overlap between the key gene modules of WGCNA and those of MEGENA. We found a large degree of overlap between the blue module and green module of WGCNA and the C1_5 module (Fig. 6A) and C1_7 (Fig. 6B) module of MEGENA. We also annotated the genes of the four modules and found that the four groups of genes are mainly involved in the cell cycle, cell division, and transcriptional functions (Fig. S7). WGCNA and MEGENA can complement each other and help us to preserve the maximum range of gene modules specific to PCa. We found that the expression profiles of this class of overlapping genes differed greatly between tumor and normal adjacent tissue, and some of the genes also differed greatly between different subtypes of tumors (Fig. 7A, B). Further, the expression of NDUFB7, AURKAIP1, ROMO1, SCAND1, GADD45GIP1, and NDUFA13 was upregulated in the four tumor subtypes compared with normal adjacent tissue (Fig. 7C). Next, we annotated the GO_BP function of 29 overlapping genes based on the DAVID database (Table 3) and found that these genes are mainly involved in cell division, cell cycle, mitosis, and positive regulation of ubiquitin-protein ligase activity (Fig. 7D).

**Table 3:** Functional annotation of GO_BP for candidate genes.

No	Term category_BP	Count	*P* value	Fold.enrichment	FDR
1	GO:0006468:protein phosphorylation	6	0.00	8.18	0.14
2	GO:0006366:transcription from RNA polymerase II promoter	5	0.00	6.06	0.64
3	GO:1904668:positive regulation of ubiquitin protein ligase activity	2	0.01	138.21	0.62
4	GO:0030071:regulation of mitotic metaphase/anaphase transition	2	0.01	138.21	0.64
5	GO:0018105:peptidyl-serine phosphorylation	3	0.02	14.93	0.64
6	GO:0,051,301:cell division	4	0.02	7.11	0.64
7	GO:0000086:G2/M transition of mitotic cell cycle	3	0.02	13.62	0.64
8	GO:0006355:regulation of transcription, DNA-templated	7	0.02	2.89	0.69
9	GO:0045862:positive regulation of proteolysis	2	0.02	65.47	0.69
10	GO:0031572:G2 DNA damage checkpoint	2	0.03	62.19	0.69
11	GO:0045736:negative regulation of cyclin-dependent protein serine/threonine kinase activity	2	0.03	56.54	0.69
12	GO:0051439:regulation of ubiquitin-protein ligase activity involved in mitotic cell cycle	2	0.05	54.08	0.69
13	GO:0045892:negative regulation of transcription, DNA-templated	4	0.04	4.99	0.74
14	GO:0000281:mitotic cytokinesis	2	0.04	42.89	0.74

### Validation of key candidate genes

**Figure 8:**
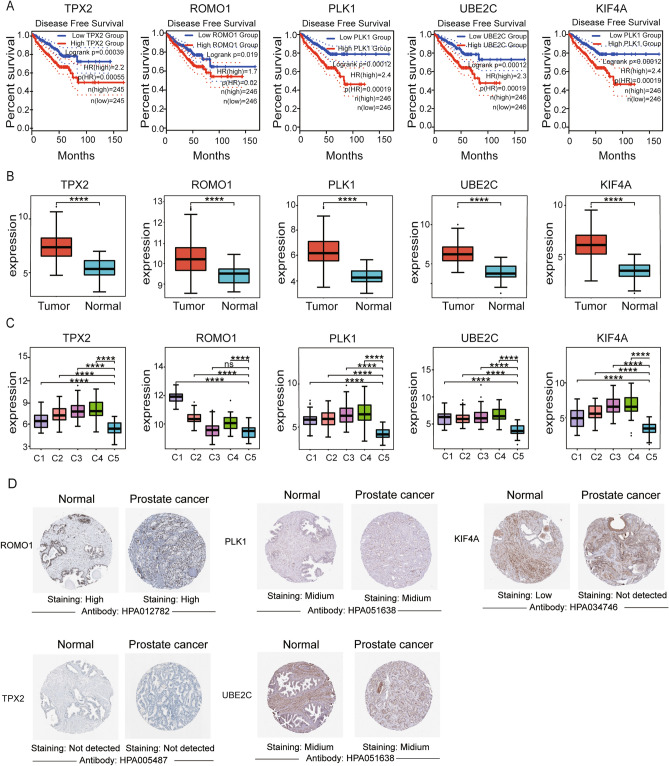
Performance of the five genes in different validation cohorts. (**A**). Prognostic value of mRNA expression of five key genes in PCa (GEPIA). PCa patients with high levels of key gene transcripts were significantly associated with short DFS. (**B**, **C**). Analysis of the expression levels of five key genes in PCa tissues and normal adjacent tissue based on the Wilcox.test method. (**C**). Analysis of the expression levels of five key genes in PCa para-cancer and four subtypes of cancer tissues based on the Wilcox.test method. The ****, ***, **, *, ns corresponding to 0, 0.001, 0.01, 0.05, 1. (**D**). Representational immunohistochemical images of ROMO1, PLK1, KIF4A, TPX2, and UBE2C in PCa and normal adjacent tissue from the HPA database. HPA, Human Protein Atlas.

We first performed a disease-free survival analysis of key genes using the GEPIA database to explore the relationship between genes and phenotype and prognostic status. We selected median as a criterion to differentiate gene expression levels and found that TPX2, ROMO1, PLK1, UBE2C, and KIF4A were considered hub genes with the most significant p-values selected from the 29 core genes (Fig. 8A). We then perform a Wilcoxon test on the five hub genes screened, and we found significant differences (*P* < 0.05) in the expression of the five genes between tumor and normal adjacent tissue (Fig. 8B). Meanwhile, we further analyzed the expression differences of the five hub genes among different tumor subtypes, and we found that these genes also differed significantly among tumor subtypes of PCa (Fig. 8C). Moreover, we found that ROMO1 was highly expressed in both tumor and normal tissues after immunohistochemistry (Fig. 8D). Meanwhile, we incorporated PCa data from GETx that found significant upregulation of ROMO1 in tumor tissues from GEPIA database (Fig. S8).

### Construction and validation of prognostic gene markers in TCGA

**Figure 9:**
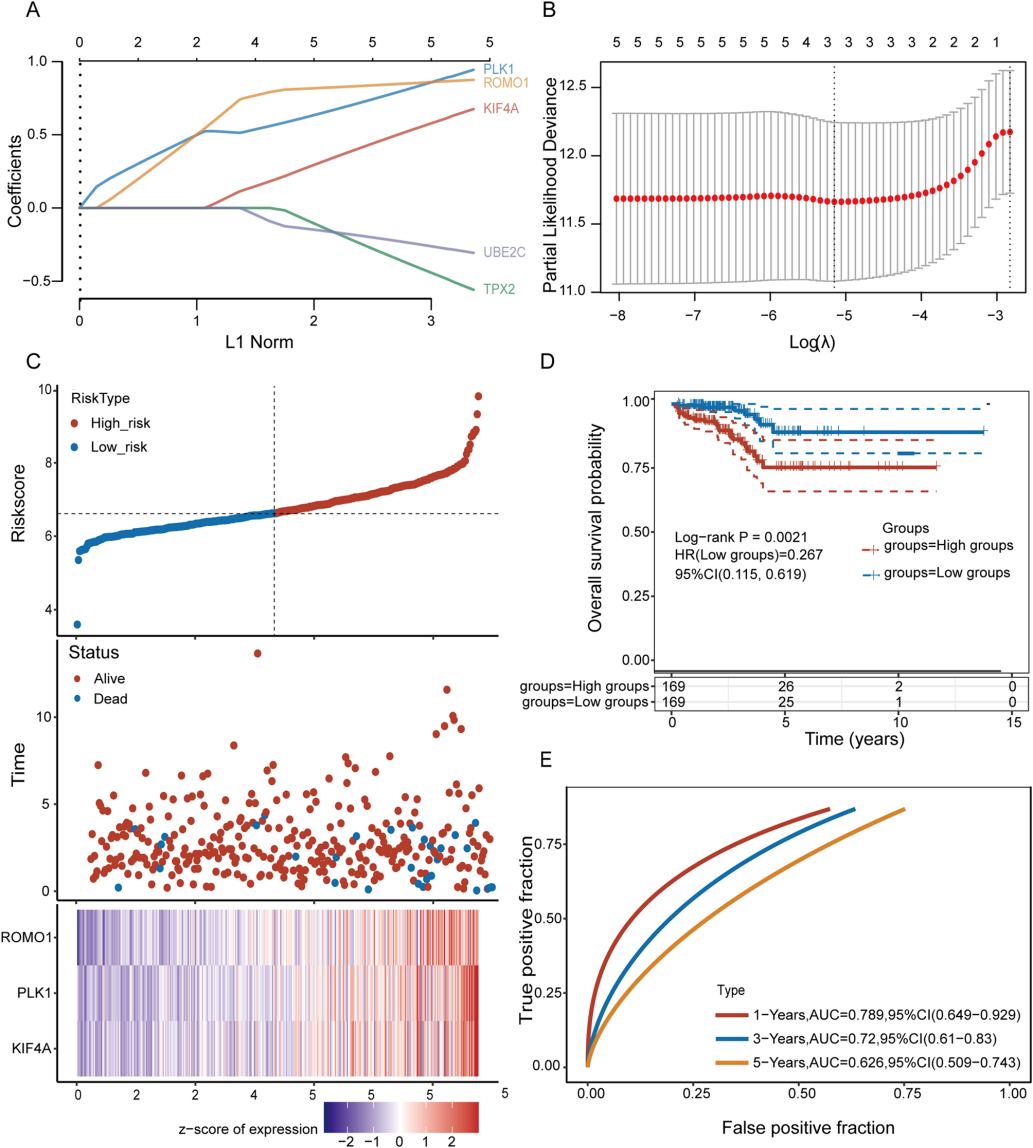
Construction of risk signature prognostic classifier in the TCGA cohort. (**A**, **B**). Determining the number of key factors through LASSO analysis. (**C**). Distribution of Pca patients based on the risk score in the TCGA database. PCA plot for OCs based on the risk score. The survival status for each patient (low-risk population: on the left side; high-risk population: on the right side). (**D**). Kaplan–Meier curves for the OS of patients in the high- and low-risk groups. (**E**). ROC curves of hub genes for predicting 1/3/5-year survival in the TCGA dataset.

The data for 497 tumors were obtained from The Cancer Genome Atlas (TCGA) dataset, and corresponding clinical information was incorporated into the prognostic training model. The expression data of five genes, TPX2, ROMO1, PLK1, UBE2C, and KIF4A, were pooled, and LASSO Cox was applied to construct a regression model. The model identified ROMO1, PLK1, and KIF4A5 based on the optimal value of λ to construct an OS prognostic model for PCa patients (Fig. 9A, B). The risk scores, patient survival status distributions, and gene expression profiles associated with the three genetic features in the training dataset are shown in Fig. 9C. OS was significantly lower in the high-risk group than in the low-risk group (*P* = 0.0021) (Fig. 9D). Finally, ROC analysis was performed, and the area under the ROC curve (AUC) for overall survival (OS) at 1, 3, and 5 years was 0.789 and 0.72 and 0.626, respectively (Fig. 9E). In a subsequent study, we also did a LASSO regression analysis on ROMO1 and found that ROMO1 could also be used as an independent prognostic model and that its low-risk group had a better survival outcome than the high-risk group (Fig. S9).

### Alterations in the immune microenvironment may result from aberrant regulation of key genes in PCa

**Figure 10:**
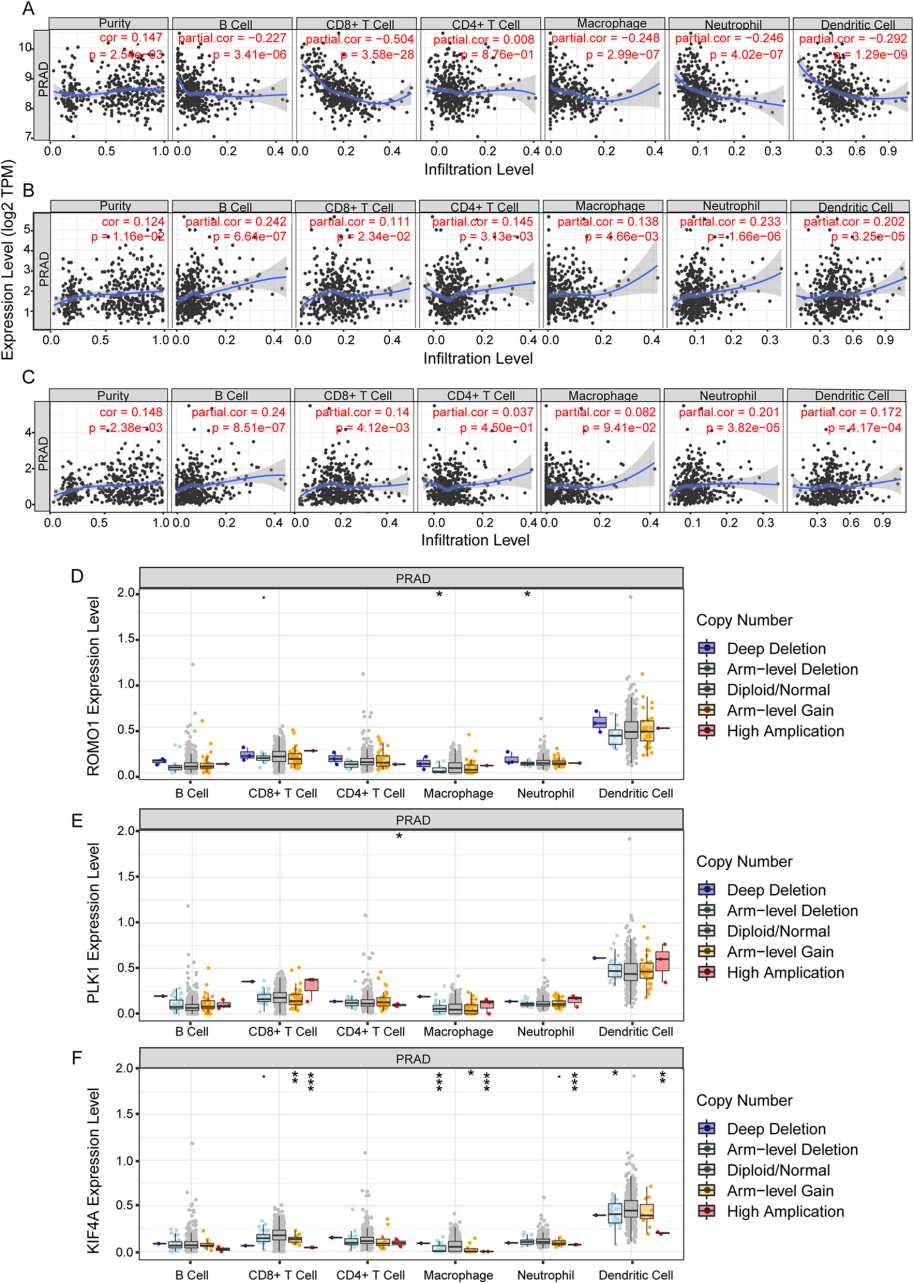
Immune relevance of three hub genes in PCa based on Tumor Immune Estimation Resource (TIMER). (**A**–**C**) Correlation study of ROMO1, PLK1 and KIF4A expression with six immune infiltrations, including CD4 + T cells, CD8 + T cells, B cells, macrophages, Neutrophils, and dendritic cells. (**D**–**F**) Boxplot shows the comparison of the tumor infiltration levels of ROMO1, PLK1 and KIF4A in immune cells with copy number variants in four categories. *P < 0.05, **P < 0.01, ***P < 0.001.

To explore the relationship between hub genes expression and tumor-infiltrating immune cells, we used the TIMER tool to analyze the correlation between key gene expression and the level of immune infiltration. Our results showed that ROMO1 expression was strongly correlated with immunity to PCa and was found to be negatively correlated with B cells, CD8 + T cells, macrophages, neutrophils, dendritic cells and positively correlated with CD4 + T cells; PLK1 and KLF4A expression was positively correlated with B cells, CD8 + T cells, macrophages, neutrophils, dendritic cells (Fig. 10 A-C). In addition, we found that different mutant forms of ROMO1 correlated with immune infiltration of CD8 + T cells, macrophages, and neutrophils; different mutant forms of KLF4A correlated with immune levels of CD8 + T cells, macrophages, neutrophils, and dendritic cells; and we found that ROMO1, PLK1, and KLF4A were found to have higher levels of immune infiltration in dendritic cells (Fig. 10 D–F). This also reveals the influence of key genes on the immune microenvironment of PCa.

### Drug sensitivity and resistance of key genes

**Figure 11:**
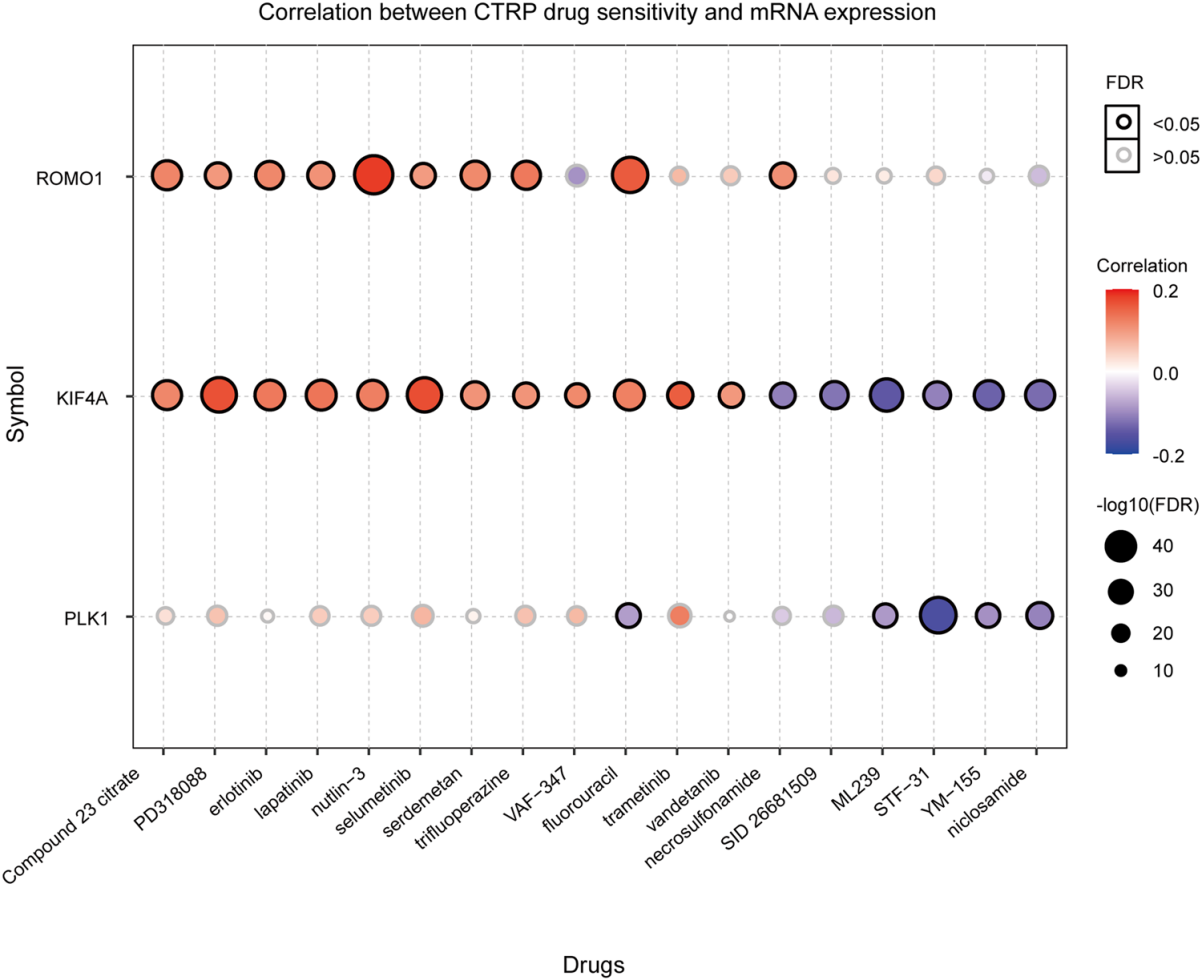
Correlation between CTRP drug sensitivity and mRNA expression.

To investigate the relationship between the expression of hub genes and drug interactions, we used the GSCA database to screen for drugs that interacted with it. The GSCA database integrated drug sensitivity and gene expression profile data for cancer cell lines from both the GDSC and CTRP databases into GSCALite, and then, using Spearman correlation analysis, the expression levels of genes were correlated with the IC50. FDR < 0.05 was considered significant (Supplementary Table 1. During the research, we found that ROMO1 expression was significantly positively correlated with nutlin-3, fluorouracil, etc. KIF4A expression was significantly positively correlated with PD318088 and selumetinib, and negatively correlated with ML239; PLK1 expression was significantly negatively correlated with STF-31 was significantly negatively correlated (Fig. 11). This reveals a diversity of expression levels of key genes and drug sensitivity.

## Discussion

In recent years, PCa has become the most common and deadly solid cancer and genitourinary tumor in men worldwide^64^, with a diagnosis rate of 12% and a mortality rate of 9%, and its incidence rises with age^65^. The traditional clinical treatment options include resection, radiotherapy, chemotherapy, and endocrine therapy^66^. The tumor heterogeneity leads to limitations in conventional treatment and makes it difficult to manage and risk assess patients effectively^67,68^. Recent studies have demonstrated the imperative of identifying new effective biomarkers and promising immune-related therapeutic targets that can be used to guide cancer treatment in clinical practice^69^. In this study, we found a high correlation between ROMO1 expression and clinical features such as the occurrence of PCa, subtypes classified by consistent clustering. Also, ROMO1 correlated with the level of immune cell infiltration and immune pathways in PCa.

Firstly, many factors can contribute to cancer development, such as dietary status, genetic mutations, epigenetic alterations, etc. We consequently identified 260 valid samples comprising a normal group and four tumor sub-types by consensus clustering and principal component analysis after outliers were excluded. Secondly, the limmaR package was selected to differentially analyze the four groups of tumor subtypes from the normal group, respectively. Finally, 3521 DEGs were identified and functionally annotated, and these differentially expressed genes were found to be mainly enriched in ah immune-related pathways. Next, WGCNA and MEGENA were applied to construct co-expression networks of differentially expressed genes to identify gene modules associated with tumorigenesis and heterogeneity, and 29 overlapping genes were identified by calculation. Then, the Lasso Cox regression model was constructed to identify ROMO1, PLK1, and KIF4A5 as the optimal core genes. Subsequently, when analyzing the relationship between the expression of hub genes and tumor-infiltrating immune cells, it was found that the expression of ROMO1, PLK1, and KIF4A were all associated with tumor-infiltrating immune cells, which in turn led to the alteration of the tumor microenvironment alterations, which in turn lead to tumor heterogeneity. Finally, we found that nutlin-3, fluorouracil, and others could be potential therapeutic agents for ROMO1, and PD318088 and selumetinib could be potential therapeutic agents for PLK1. In conclusion, our study provides a new theoretical basis for the diagnosis and treatment of PCa.

Polo-like kinase 1 (PLK1) is a member of a family of serine/threonine protein kinases that are widely found in eukaryotic cells^70^. Its specific functions are mainly in cell cycle processes, including controlling mitotic entry and the G2/M checkpoint, coordinating centrosomes and the cell cycle, regulating spindle assembly and chromosome segregation, performing multiple functions during mid-spindle and abscission, promoting DNA replication, participating in the cytoplasmic division and meiosis, and playing an important role in the initiation, maintenance, and completion of mitosis^71–73^. The pharmacological inhibition of PLK1 in triple-negative breast cancer has been reported to increase the anti-proliferative activity of drug-resistant cells, which in turn causes G2/M phase block and increases the phosphorylation of cell cycle proteins inducing apoptosis^74^. Also, in PCa, mitotic kinase polo-like kinase 1 (PLK1) is expressed at elevated levels and is associated with tumor grade^75^. In the present study, we also found a significant prognostic effect of PLK1, with expression in different subtypes of the prostate gland also differing significantly from normal samples, and found that PLK1 expression was also associated with tumor-infiltrating immune cells, further demonstrating the reliability of PLK1 as a biomarker for PCa.

Kinesin superfamily protein 4A (KIF4A) is found in all eukaryotes and belongs to a family of KIFs that are highly conserved^76^. KIF4A has important roles in DNA repair, DNA replication, spindle organization, cytoplasmic division, and intracellular transport^77^. KIF4A has been previously reported to be aberrantly expressed in many cancers, revealing its function and role in different tumors^78–80^. KIF4A can promote PCa cell growth through AR and AR-V7-dependent signaling^78^. In the present study, KIF4A expression in different subtypes of the prostate was also significantly different from normal samples, and KIF4A expression was also found to be associated with tumor-infiltrating immune cells.

Studies have shown that ROMO1 is overexpressed in hepatocellular carcinoma, colorectal cancer, and glioma^81–83^ but has not been reported in PCa. In the present study, we selected the Wilcoxon test and found that ROMO1 was highly expressed in tumor tissue and significantly different from normal tissue; we also found that the four identified tumor subtypes were significantly different. The expression of ROMO1 was also found to be associated with tumor-infiltrating immune cells, leading to changes in the tumor microenvironment and further increasing tumor heterogeneity; drug sensitivity analysis revealed that nutlin-3 and fluorouracil could be used as potential therapeutic agents for ROMO1.

In general, tumor pathogenesis involves many interacting signaling pathways, including tumor cell proliferation, cell immortalization, invasion, and migration, etc.^84^. The complexity of cancer can be reflected through the tumor microenvironment, while protein interactions can further increase heterogeneity between tumors^85,86^. In the present study, we have used weighted gene co-expression network analysis (WGCNA), which classifies the gene co-expression network of PCa into ten highly correlated signature modules. The modules were then correlated with specific clinical features to identify genes that are key to tumorigenesis and transformation, to help identify potential mechanisms involved, and to explore candidate biomarkers. However, WGCNA has the limitation of not being able to coexist at different levels of clustering within a single network, thus not reflecting the multi-scale hierarchical nature of complex networks. Multi-scale embedded gene co-expression network analysis (MEGENA), on the other hand, allows the construction and analysis of large-scale planar filtered co-expression networks to the greatest extent possible^53^. Parallelization of embedded network construction and the identification of multiscale clustering structures are two key components of MEGENA, which is an essential complement to existing co-expression network analysis methods by identifying multiscale modular systems and co-expression networks with varying degrees of sparse and tight connectivity. Here, by using WGCNA in conjunction with MEGENA to construct a gene co-expression network for PCa, we identified more meaningful clusters of co-expressed genes and identified key biomarkers associated with prostate carcinogenesis in transformation.

In this study, we combined various bioinformatic analysis methods, especially the introduction of weighted gene co-expression network analysis and multi-scale chimeric network analysis, to reveal that ROMO1 may serve as a new key prognostic marker for PCa. However, the article still has some limitations. Firstly, the role of ROMO1 has not been validated experimentally in vivo and in vitro, and secondly, the fact that the number of cancer samples and normal samples are not identical to each other has led to some preference in our data. We believe that if we had direct access to a larger sample of clinical sequencing data and sample information, we would obtain better and more accurate results.

## Conclusions

In conclusion, our systematic analysis of the TCGA database supported by array-based and sequence-based PCa data has identified ROMO1, a key gene closely associated with the PCa tumor microenvironment, and the essential signaling pathways involved. For this reason, we propose that ROMO1 may serve as a potential biomarker and therapeutic target for PCa. It may provide new theoretical inferences for the diagnosis and prognosis of PCa.

## Supplementary Information

Supplementary Table 1.

Supplementary Figures.

## Data Availability

All data are available. Please contact us to access if it is needed.

## Abbreviations

PCa: Prostate cancer
WGCNA: Weighted Gene Co-expression Network Analysis
MEGENA: Multiscale Embedded Gene Co-expression Network Analysis
DEG: Differentially expressed genes
PSA: Serum prostate-specific antigen
DFS: Disease-free survival
PFN: Planar filtered network
MCA: Multiscale Clustering Analysis
HPA: The Human Protein Atlas
MHA: Multiscale hub analysis
CTA: Cluster-trait association analysis
GS: Gene TraitSignificance
MM: Module-Membership
IHC: Immunohistochemistry
TCGA: The cancer genome atlas
GO: Gene ontology
LASSO: Least absolute shrinkage and selection operator
GEPIA: Gene expression profling interactive analysis
AUC: Area under curve
TIMER: Tumor Immunity Evaluation
OS: Resource overall survival
ROC: Receiver operating characteristic
GSEA: Gene set enrichment analysis
