# Supplementary material for: Network models of prostate cancer immune microenvironments identify ROMO1 as heterogeneity and prognostic marker
Source: Sci Rep. 2022 Jan 7;12:192. doi: 10.1038/s41598-021-03946-w (PMC8741951; doi:10.1038/s41598-021-03946-w)
Supplement: Supplementary file 1 — Supplementary Table 1. [file 41598_2021_3946_MOESM1_ESM.docx]

**Supplementary Table1. Drugs that interact with the hub gene.**

| **NO.** | **SYMBOL** | **DRUE** | **COR** | **FDR** |
| --- | --- | --- | --- | --- |
| 1 | KIF4A | Compound 23 citrate | 0.12 | 0.00 |
| 2 | KIF4A | ML239 | -0.15 | 0.00 |
| 3 | KIF4A | PD318088 | 0.17 | 0.00 |
| 4 | KIF4A | SID 26681509 | -0.12 | 0.01 |
| 5 | KIF4A | STF-31 | -0.11 | 0.01 |
| 6 | KIF4A | VAF-347 | 0.12 | 0.05 |
| 7 | KIF4A | YM-155 | -0.14 | 0.00 |
| 8 | KIF4A | erlotinib | 0.13 | 0.00 |
| 9 | KIF4A | fluorouracil | 0.12 | 0.00 |
| 10 | KIF4A | lapatinib | 0.14 | 0.00 |
| 11 | KIF4A | necrosulfonamide | -0.11 | 0.02 |
| 12 | KIF4A | niclosamide | -0.13 | 0.00 |
| 13 | KIF4A | nutlin-3 | 0.13 | 0.00 |
| 14 | KIF4A | selumetinib | 0.17 | 0.00 |
| 15 | KIF4A | serdemetan | 0.11 | 0.01 |
| 16 | KIF4A | trametinib | 0.16 | 0.02 |
| 17 | KIF4A | trifluoperazine | 0.12 | 0.02 |
| 18 | KIF4A | vandetanib | 0.10 | 0.03 |
| 19 | PLK1 | Compound 23 citrate | 0.03 | 0.47 |
| 20 | PLK1 | ML239 | -0.08 | 0.05 |
| 21 | PLK1 | PD318088 | 0.06 | 0.23 |
| 22 | PLK1 | SID 26681509 | -0.06 | 0.23 |
| 23 | PLK1 | STF-31 | -0.17 | 0.00 |
| 24 | PLK1 | VAF-347 | 0.07 | 0.28 |
| 25 | PLK1 | YM-155 | -0.09 | 0.044 |
| 26 | PLK1 | erlotinib | 0.01 | 0.91 |
| 27 | PLK1 | fluorouracil | -0.08 | 0.04 |
| 28 | PLK1 | lapatinib | 0.05 | 0.29 |
| 29 | PLK1 | necrosulfonamide | -0.04 | 0.41 |
| 30 | PLK1 | niclosamide | -0.10 | 0.016 |
| 31 | PLK1 | nutlin-3 | 0.05 | 0.25 |
| 32 | PLK1 | selumetinib | 0.07 | 0.14 |
| 33 | PLK1 | serdemetan | 0.01 | 0.81 |
| 34 | PLK1 | trametinib | 0.13 | 0.07 |
| 35 | PLK1 | trifluoperazine | 0.06 | 0.24 |
| 36 | PLK1 | vandetanib | 0.00 | 0.99 |
| 37 | ROMO1 | Compound 23 citrate | 0.12 | 0.00 |
| 38 | ROMO1 | ML239 | 0.02 | 0.71 |
| 39 | ROMO1 | PD318088 | 0.10 | 0.02 |
| 40 | ROMO1 | SID 26681509 | 0.02 | 0.63 |
| 41 | ROMO1 | STF-31 | 0.04 | 0.38 |
| 42 | ROMO1 | VAF-347 | -0.09 | 0.13 |
| 43 | ROMO1 | YM-155 | -0.02 | 0.78 |
| 44 | ROMO1 | erlotinib | 0.12 | 0.00 |
| 45 | ROMO1 | fluorouracil | 0.16 | 0.00 |
| 46 | ROMO1 | lapatinib | 0.11 | 0.01 |
| 47 | ROMO1 | necrosulfonamide | 0.11 | 0.02 |
| 48 | ROMO1 | niclosamide | -0.05 | 0.23 |
| 49 | ROMO1 | nutlin-3 | 0.18 | 2.01 |
| 50 | ROMO1 | selumetinib | 0.10 | 0.03 |
| 51 | ROMO1 | serdemetan | 0.12 | 0.00 |
| 52 | ROMO1 | trametinib | 0.07 | 0.39 |
| 53 | ROMO1 | trifluoperazine | 0.13 | 0.00 |
| 54 | ROMO1 | vandetanib | 0.05 | 0.32 |
