# Supplementary material for: Network models of prostate cancer immune microenvironments identify ROMO1 as heterogeneity and prognostic marker
Source: Sci Rep. 2022 Jan 7;12:192. doi: 10.1038/s41598-021-03946-w (PMC8741951; doi:10.1038/s41598-021-03946-w)
Supplement: Supplementary file 2 — Supplementary Figures. [file 41598_2021_3946_MOESM2_ESM.docx]

**supplementary material section**

**
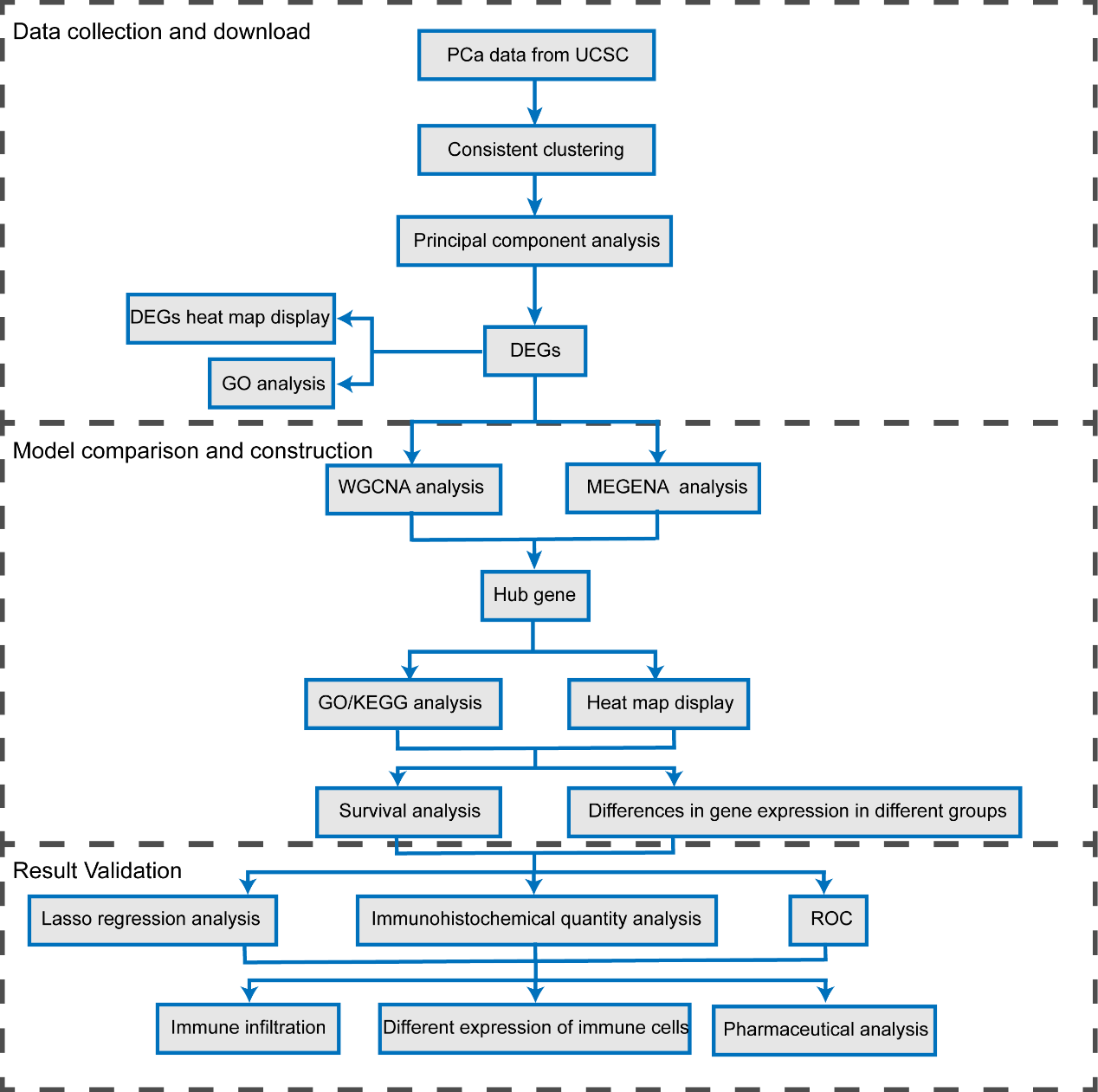
**

**Figure S1. Flow chart of the technical route of this study.**


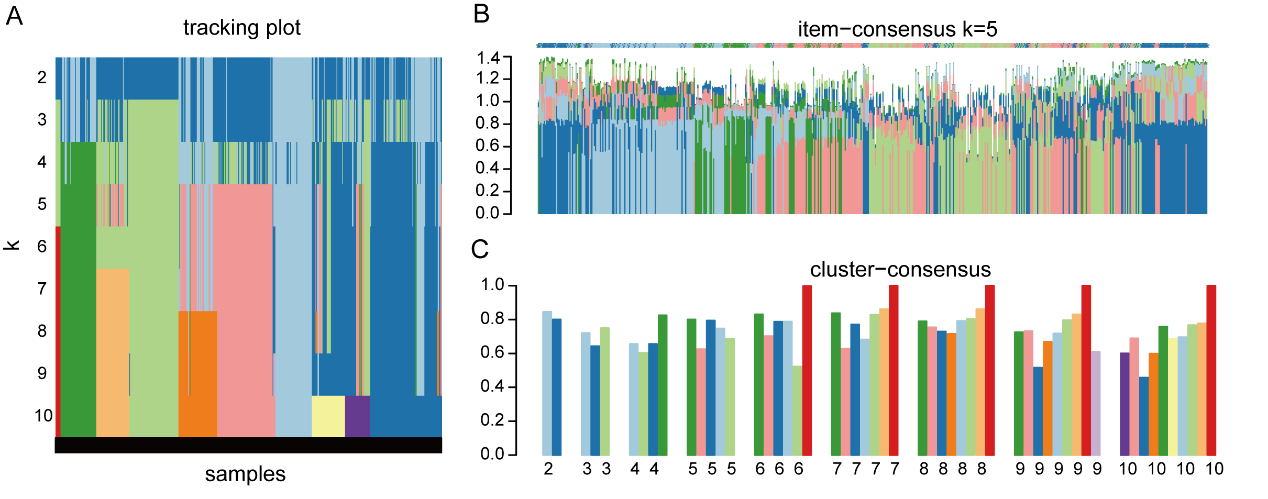


**Figure S2. Determine the suitable k value for consensus clustering.** (A). Trace plots of samples from k=2 to k=9. (B). Item-consensus plot of IC value of the item (item -consensus, IC) at k=5. IC chart shows items as vertical bars of colored rectangles whose height corresponds to the IC value. (C). Cluster-consensus plot (CLC) evaluate the impact of adding new clusters on the CLC values of existing cluster


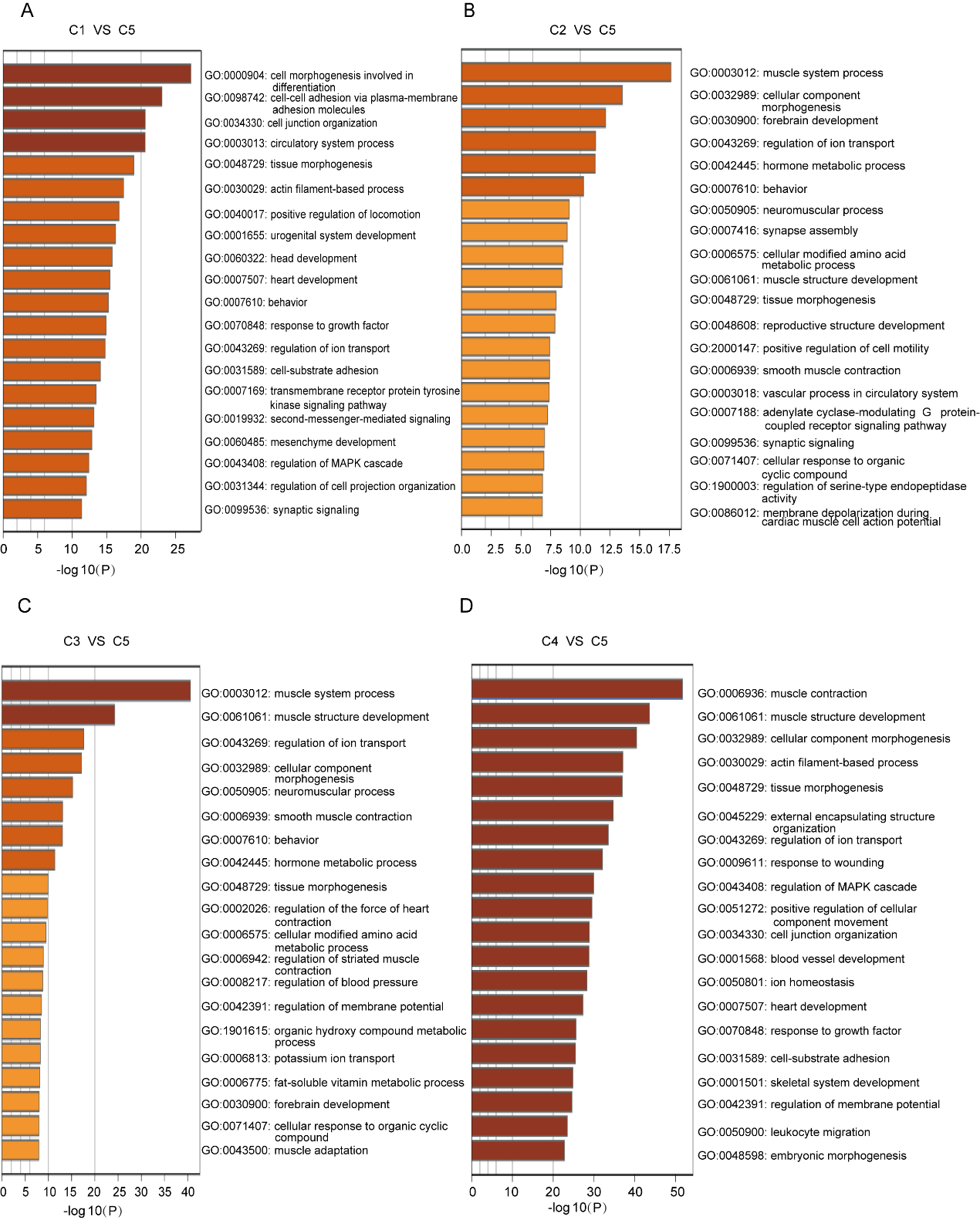


**Figure S3. GO annotation of different tumor molecular subtypes and normal tissues from Metascape website.**
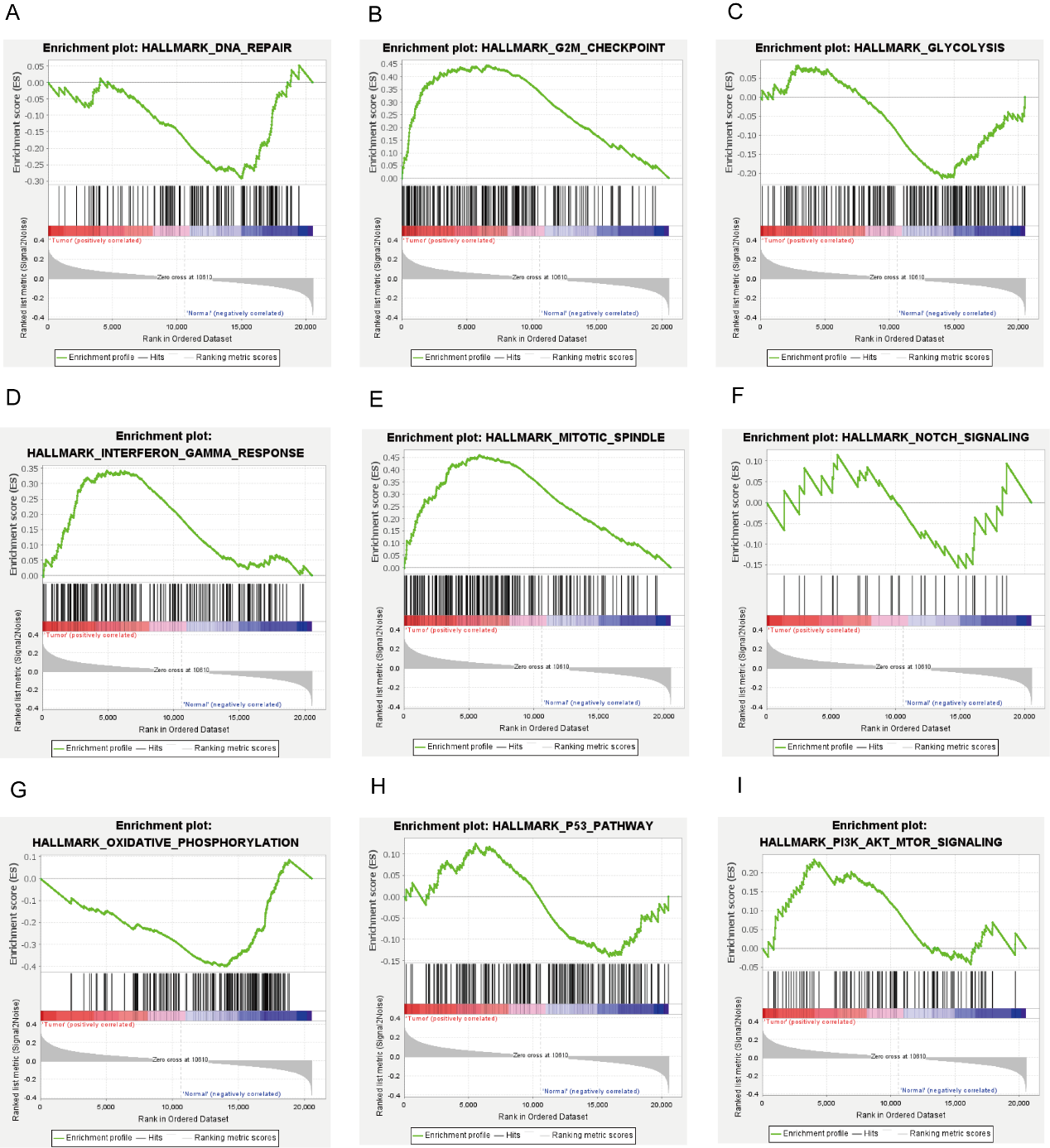


**Figure S4. gene set enrichment analysis (GSEA) showed that it was mainly enriched in PCa-related pathways. GSEA results show that p53, notch signaling pathway etc, are differentially enriched in PCa. Enrichment fraction; ES, enrichment score; NES, normalized ES; NOM p-val, normalized p-value.**


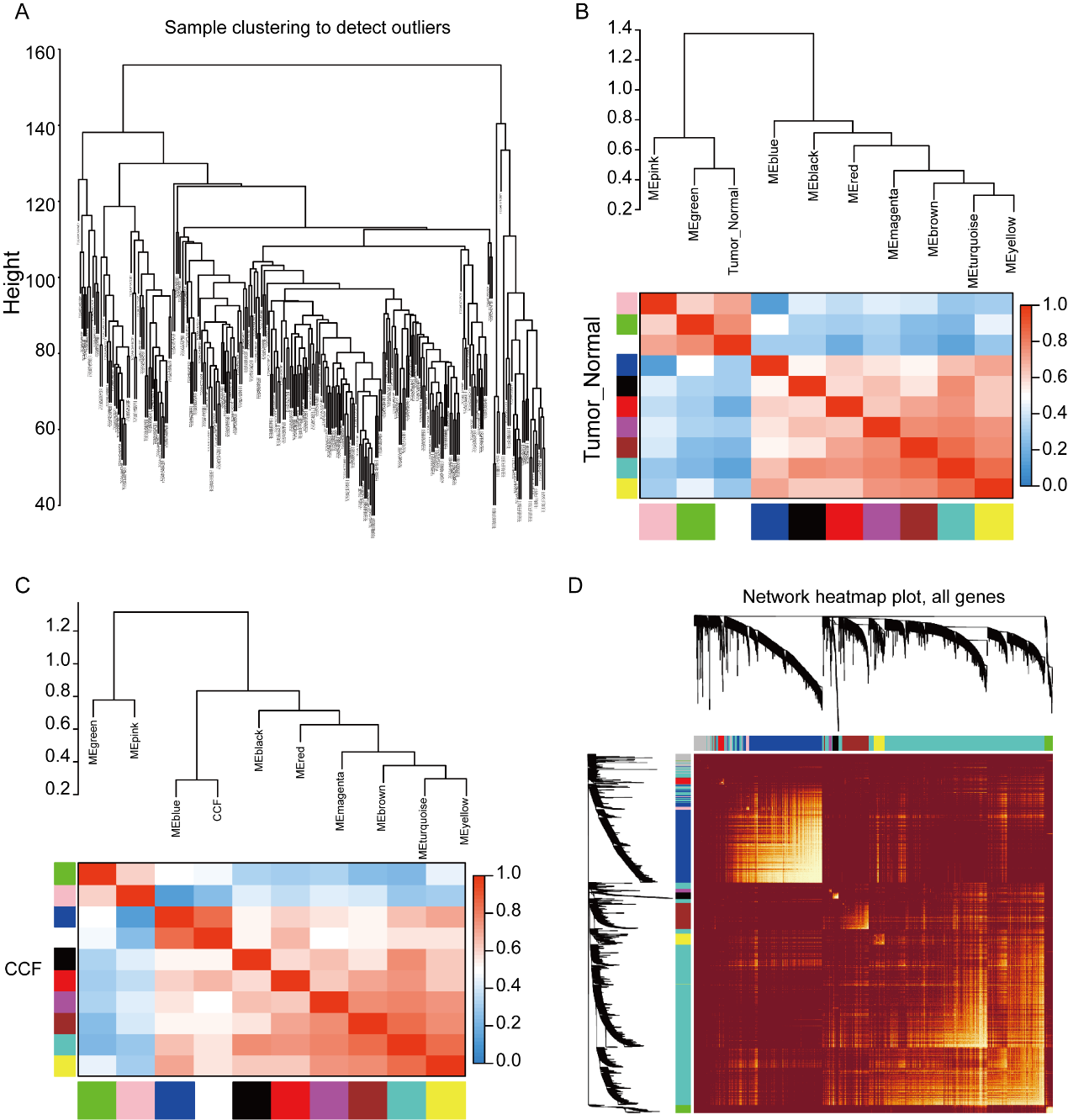


**Figure S5. WGCNA sample and module selection.**

(A)Tree diagram of sample level clustering results for prostate cancer expression data. (B-C) A dendrogram of consistent module trait genes with consistent correlations to Tumor_Normal and CCF traits with a heatmap of module neighbors. (D). Heat map of all module genes and correlation between modules.


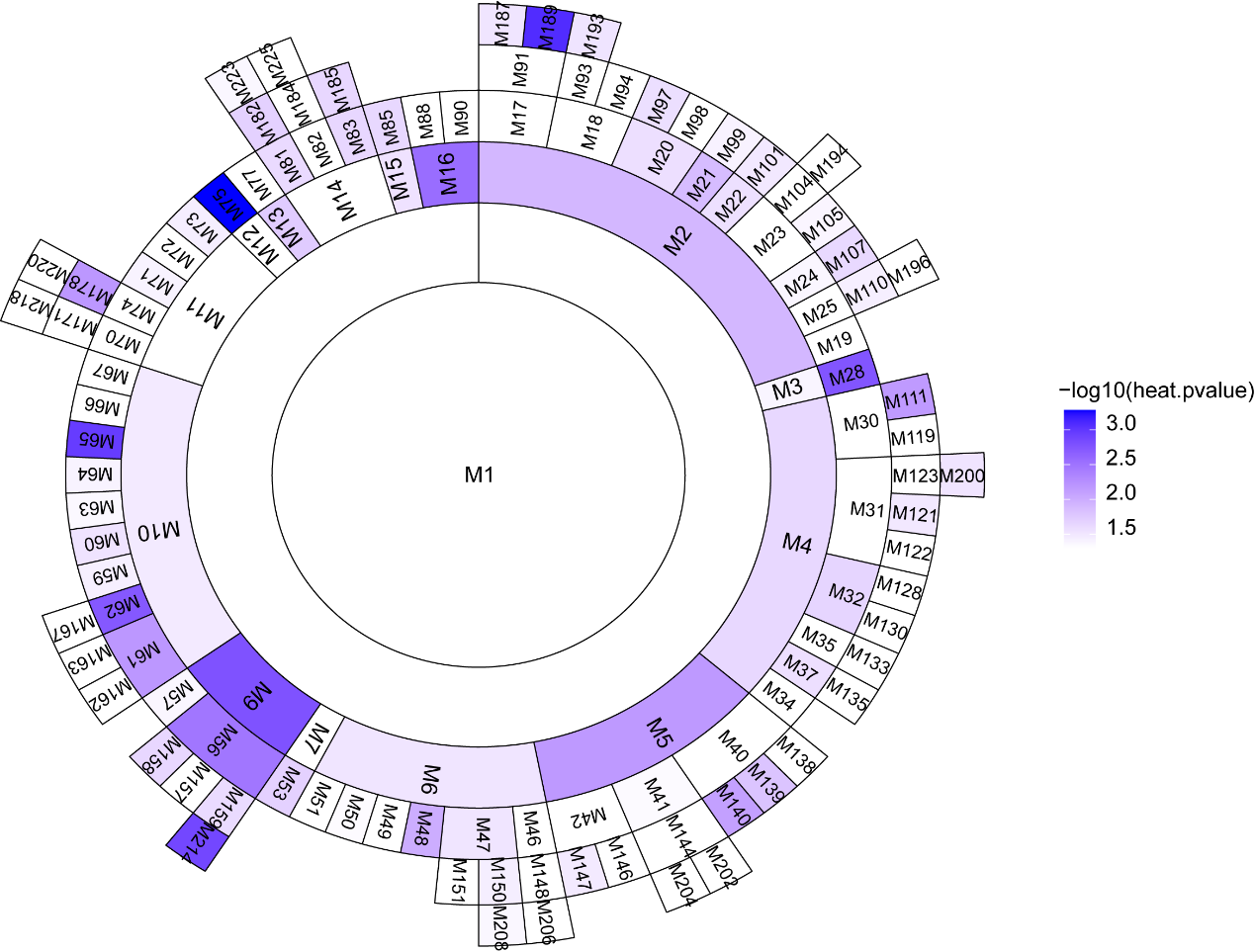


**Figure S6.** **Gene Module Construction Model for MEGENA.**


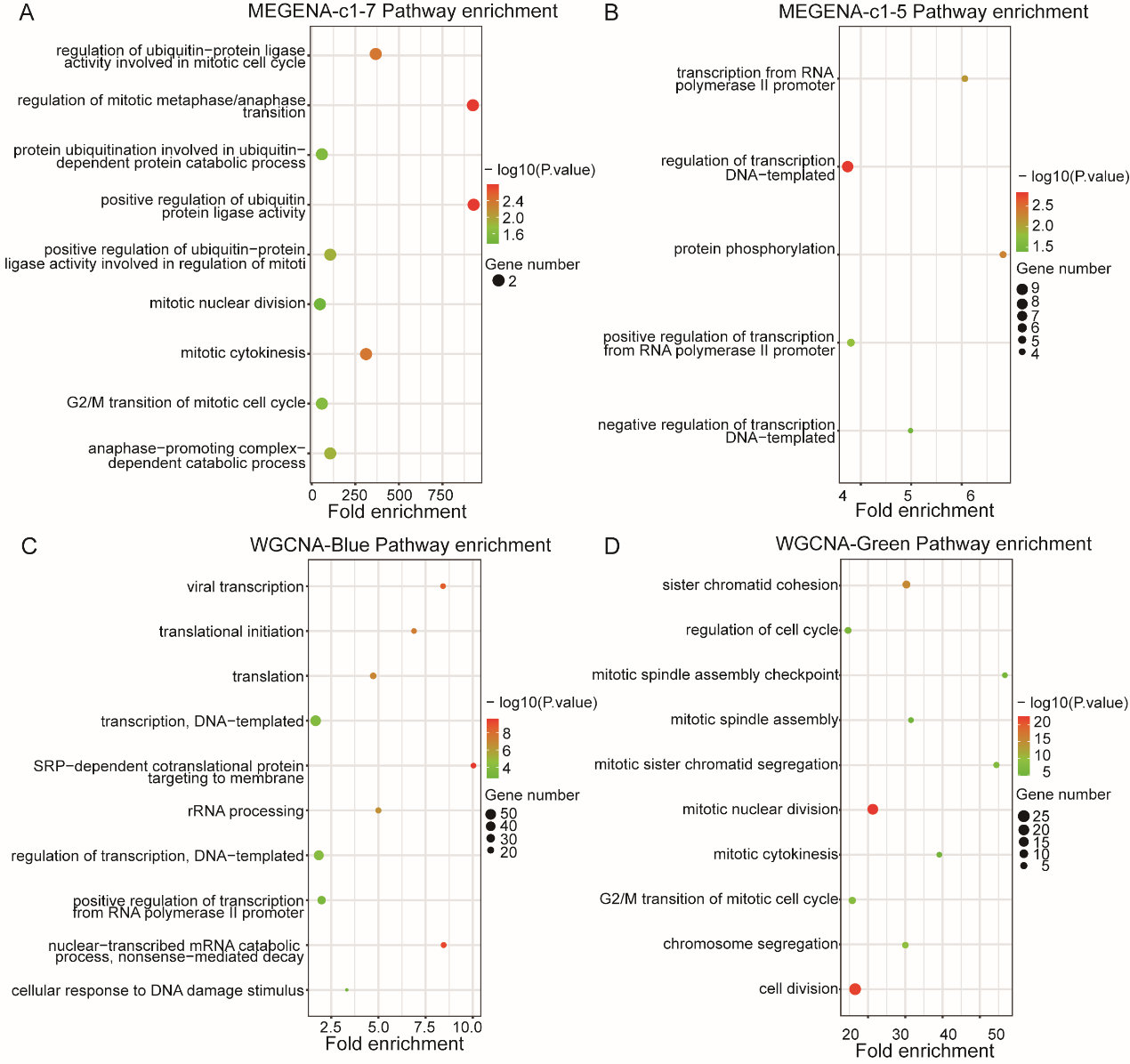


**Figure S7.** **GO functional annotation of 4 key gene modules.**


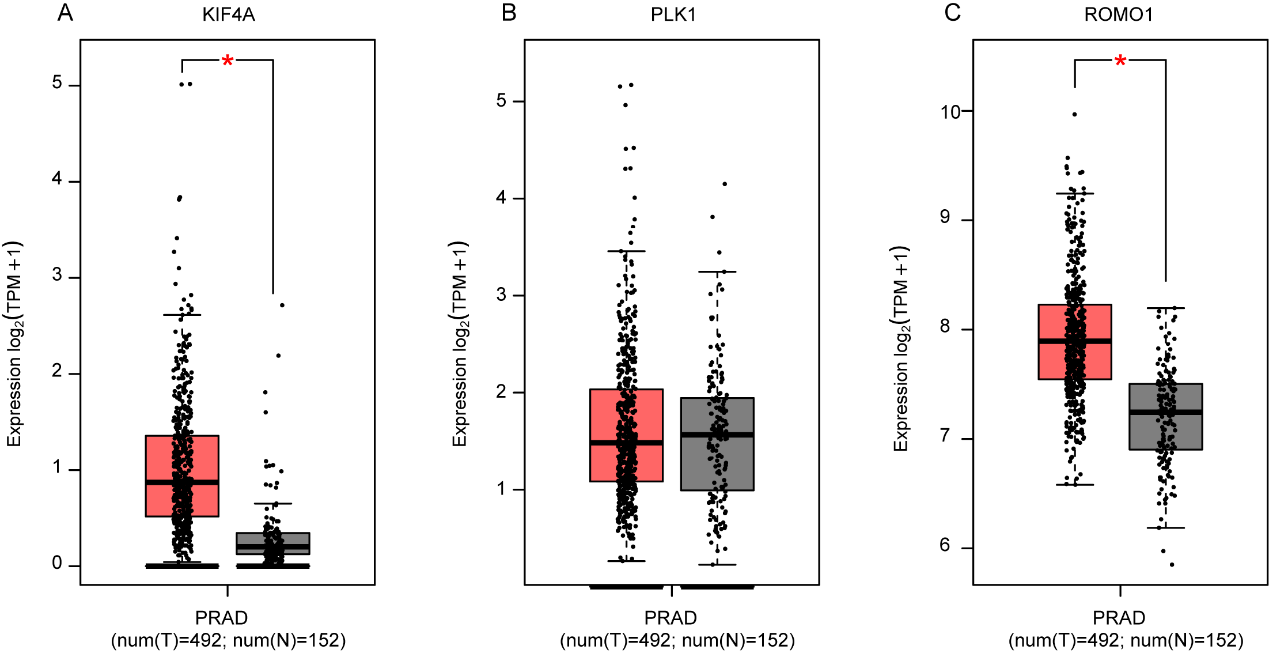


**Figure S8. Validation of hub genes from GEPIA database.**

**
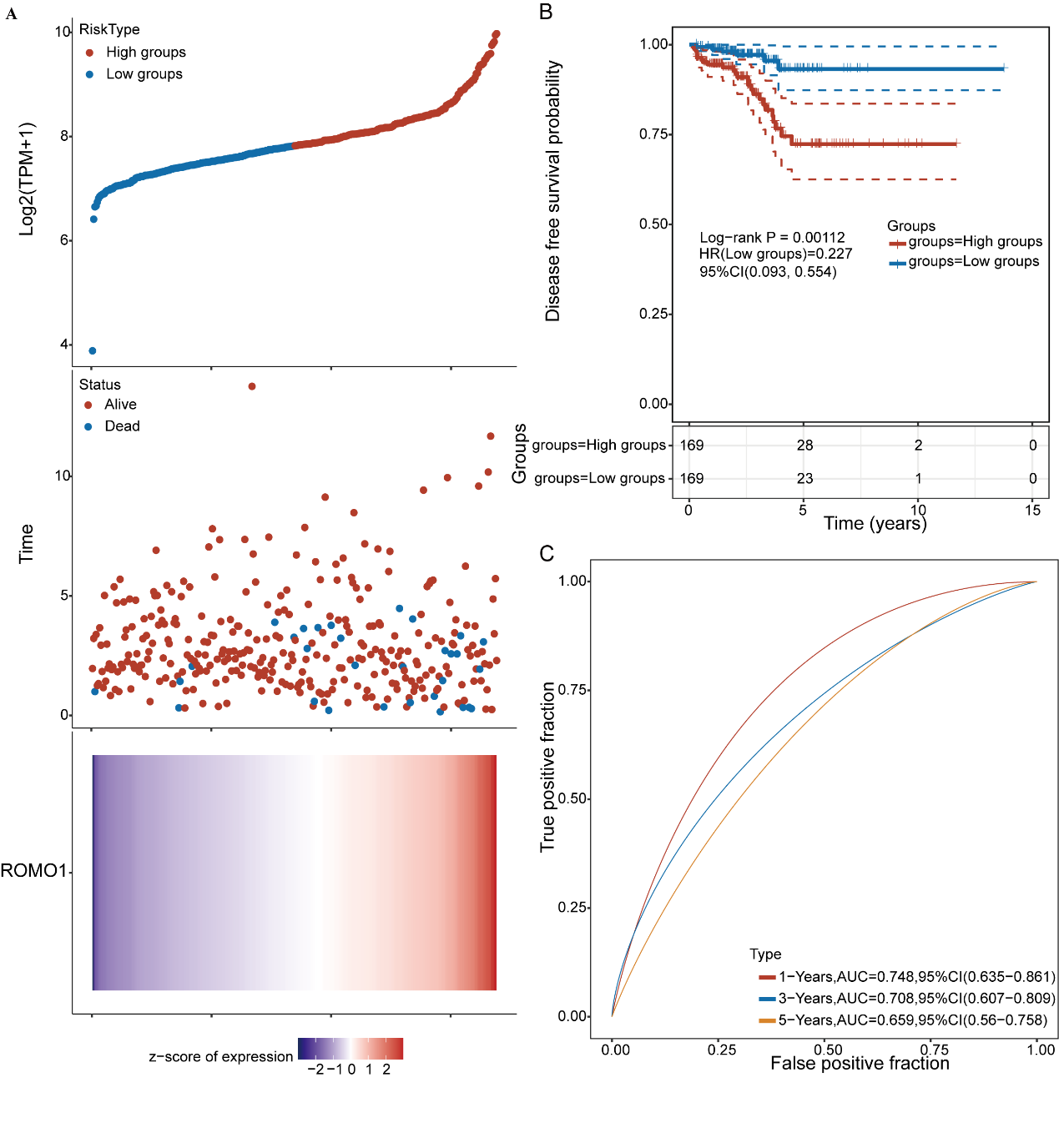
Figure S9. Construction of ROMO1 prognostic classifier in the TCGA cohort.**
